# Hybrid Series of Carbon‐Vacancy Electrodes for Multi Chemical Vapors Diagnosis Using a Residual Multi‐Task Model

**DOI:** 10.1002/advs.202500412

**Published:** 2025-05-11

**Authors:** Tianci Liu, Yun Ji Hwang, Lu Zhang, Jongwoo Hong, Teajong Hwang, Seong Chan Jun

**Affiliations:** ^1^ School of Mechanical Engineering Yonsei University 50, Yonsei‐ro, Seodaemun‐gu Seoul 03722 Republic of Korea; ^2^ School of Electrical and Electronic Engineering Yonsei University 50, Yonsei‐ro, Seodaemun‐gu Seoul 03722 Republic of Korea

**Keywords:** deep leaning, gas sensors, graphene oxides, metal oxides, multi‐task classification, sensor arrays

## Abstract

Detecting individual gases with various sensors is a well‐established field in gas sensing. However, substantial challenges and opportunities remain in the simultaneous detection and classification of multiple gases. Artificial intelligence (AI) integrated gas sensor systems effectively enable multi‐gas detection using specialized algorithms. Nevertheless, these algorithms are prone to overfitting owing to their high model complexity; this study proposes a sensor array that engineers carbon vacancies in graphene oxide via metal ion doping and high‐temperature reduction, enabling high‐sensitivity, simultaneous detection of various gases at low temperatures (20 °C). By integrating an advanced artificial intelligence framework, the acquired electrical signals are transformed, and a multi‐task learning (MTL) approach is applied to achieve instantaneous identification of four gas types and four‐level concentrations. The proposed MTL framework demonstrates superior performance by effectively mitigating overfitting and improving generalization through feature sharing and mutual regularization between gas type classification and concentration estimation tasks. Experimental validation on vehicle exhaust gas fault diagnosis highlights the method's effectiveness and applicability in complex conditions, achieving 98.22% accuracy and 48% faster inference compared to traditional single‐task models. This study provides a basis for developing more intelligent and adaptable sensor systems capable.

## Graphical abstract

This study presents a graphene oxide‐based gas sensor array integrated with a deep learning framework for precise detection and classification of multiple gases. By combining advanced sensing materials and a 1D‐ResNet architecture, the system achieves high sensitivity and accuracy, demonstrating its potential for real‐time environmental monitoring, industrial safety, and vehicle exhaust analysis.

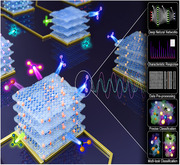

## 1 Introduction

The rapid expansion of global industry has substantially increased environmental pollutants, particularly air contaminants, posing severe risks to human health and the overall quality of life^[^ ^1^, ^2^ ^]^ A substantial challenge in gas detection is identifying harmful gases at low concentrations, which is crucial for public safety, environmental monitoring, health diagnostics, and industrial applications.^[^ ^2^, ^3^, ^4^ ^]^ Although traditional techniques, such as gas chromatography (GC)^[^ ^5^ ^]^ and mass spectrometry,^[^ ^6^ ^]^ have been demonstrated to be reliable for gas detection and identification, they involve complex instrumentation, cumbersome procedures, and high maintenance costs, limiting their practicality for routine monitoring.^[^ ^2^, ^7^ ^]^

Gas sensors are emerging as promising alternatives because of their low cost, small size, and real‐time monitoring ability.^[^ ^8^ ^]^ Unfortunately, state‐of‐the‐art gas sensors often exhibit high cross‐sensitivity and poor selectivity,^[^ ^9^ ^]^ rendering it challenging for standalone sensors to accurately identify multiple gases with sufficient accuracy.^[^ ^10^ ^]^ Conventional metal oxide (MO) sensors and their composites are especially prone to these shortcomings. To overcome these challenges, researchers have explored various strategies to enhance sensor performance, including controlling particle size and shape, optimizing porosity, and modifying surfaces.^[^ ^11^ ^]^ Among these approaches, metal atom doping and vacancy engineering have emerged as particularly effective techniques. Oxygen vacancies are typically produced by transition metal substituents with different valence state. By introducing impurity levels or defect band centers, these methods enable fine‐tuning of the electronic structure, thereby improving ionic conductivity and optimizing adsorption/desorption characteristics for target analytes.^[^ ^12^ ^]^ Despite these advances, one major drawback of metal oxide–based sensors is their need for high operating temperatures, which restricts their practical applications and limits their versatility across diverse environments. Furthermore, the high cost and necessity for regular maintenance and calibration increase the complexity and expense of utilizing such systems.^[^ ^13^ ^]^

Overcoming the challenges associated with integrating algorithms and sensor arrays is a pivotal advancement in gas classification and identification. However, achieving this integration remains extremely challenging.^[^ ^14^ ^]^ Multielectrode sensor arrays capable of simultaneously outputting multiple response signals have attracted substantial attention as a cost‐effective and versatile sensor technology.^[^ ^15^ ^]^ Despite their versatility, traditional multielectrode sensor arrays often face substantial limitations.^[^ ^16^ ^]^ One of the primary challenges is cross‐sensitivity, where multiple electrodes respond similarly to the same analyte, leading to a lack of signal independence. This redundancy can cause increased data overlap, reducing the effectiveness of sensor arrays in detecting and differentiating between multiple gases. It also diminishes the overall sensitivity and accuracy of the detection system, particularly in complex gas mixtures.^[^ ^16^, ^17^ ^]^ Additionally, the data generated by each sensor in the array are of paramount importance in developing artificial intelligence models for gas identification.^[^ ^18^ ^]^ For instance, deep learning techniques, such as convolutional neural networks (CNNs),^[^ ^19^ ^]^ recurrent neural networks (RNNs),^[^ ^20^ ^]^ and graph neural networks(GNNs),^[^ ^21^ ^]^ enable automatic extraction and learning of intricate features. This considerably improves the accuracy and sensitivity of gas detection, facilitating efficient interpretation of the numerous response signals generated by the sensor array.

In this study, we present a sensor array based on graphene oxide (GO). By introducing trace amounts of metal ions (Fe^3^⁺, Zn^2^⁺, Sn^2^⁺, Ag⁺) and employing high‐temperature reduction to form metal oxides while simultaneously creating carbon vacancies that modulate the electronic structure. These modifications enhance ionic conductivity and optimize the adsorption and desorption properties of the GO surface, thereby improving specificity and sensitivity to different gases. Furthermore, each electrode in this design serves as a substrate for a distinct sensing material, enabling the array to generate independent response signals upon exposure to various gas analytes. This innovation effectively mitigates the cross‐sensitivity issue and substantially improves detection capabilities, enabling simultaneous high‐precision detection of multiple gases at significantly lower temperatures (≈20 °C) compared to conventional metal oxide sensors. The optimized reduced graphene oxide (rGO)/trace metal oxide (tMOs) sensor array design achieves an ≈200% improvement in sensing performance over pristine rGO‐based sensors. This feature effectively mitigates the cross‐sensitivity issue by providing a diverse set of response signals that are crucial for distinguishing between the various gas species. The independent and complementary nature of the responses substantially enhances the detection capabilities of the sensor array, enabling the simultaneous detection of a wide range of gas molecules with higher precision and reduced data redundancy. Unlike traditional metal oxide sensors that typically require high operational temperatures exceeding 300 °C, the proposed rGO/tMO sensor array operates effectively at a low working temperature of just 20 °C. In addition, by doping with metal ions to introduce carbon vacancies and optimizing the sensor array, the sensor's performance improved by nearly 200% compared to the original rGO sensor. This low‐temperature operation reduces energy consumption and minimizes the thermal degradation of sensitive components, effectively addressing the typical limitation of conventional high‐temperature gas detection methods. The uniform low‐temperature operation across all nine electrodes ensured consistent sensor performance and enhanced the reliability of the sensor system.

Furthermore, a deep learning network was also utilized to enhance data analysis capabilities. Building on this, a multi‐task learning classification framework was applied for the first time in the field of gas sensing. This framework incorporates multiple distinct label columns to classify various outcomes independently, enabling high‐precision, simultaneous identification of both gas types and concentrations. Moreover, a classic single‐label column precision classification task was implemented to further refine the experimental results and provide a benchmark reference for multitask classification performance. The introduction of this novel multi‐task learning (MTL) approach allows joint feature extraction and mutual regularization between tasks, significantly improving generalization performance and effectively reducing overfitting issues commonly seen in complex classification scenarios. This approach enabled outstanding sub‐ppm detection and differentiation of four harmful gases: carbon monoxide(CO), nitrogen dioxide(NO_2_), nitric oxide(NO), and ethanol(C_2_H_5_OH). Finally, to validate the sensor's recognition performance in practical applications, simulation experiments were conducted using complex vehicle exhaust gases, including diesel and gasoline exhaust diluted to various degrees. In addition, four typical engine fault conditions were simulated to assess the ability of the sensor to detect and classify different operational scenarios. These real‐world experimental validations confirm the practical applicability and reliability of our sensor‐AI integrated system, demonstrating a classification accuracy of 100% for exhaust gas types, ≈98% accuracy for combined gas type‐concentration identification, and robust detection of engine fault conditions with an accuracy of 98.22%. The results demonstrated the classification of two distinct exhaust gas types with 100% accuracy. Furthermore, the multi‐classification system exhibited an accuracy of 98% for the precise identification of the concentration and type of gases. Moreover, the detection and identification of the engine faults demonstrated an accuracy of 98.22%. These results underscore the capability of the proposed sensor, in combination with the developed algorithmic model, to accurately identify complex gas mixtures, even at low concentrations. This advancement has substantial potential for revolutionizing environmental monitoring, optimizing vehicle emission control, enhancing industrial safety, and paving the way for more intelligent and effective sensing systems.

## 2 Results

### 2.1 Deep Learning‐Based Multi‐Gas Sensing System

**Figure 1:**
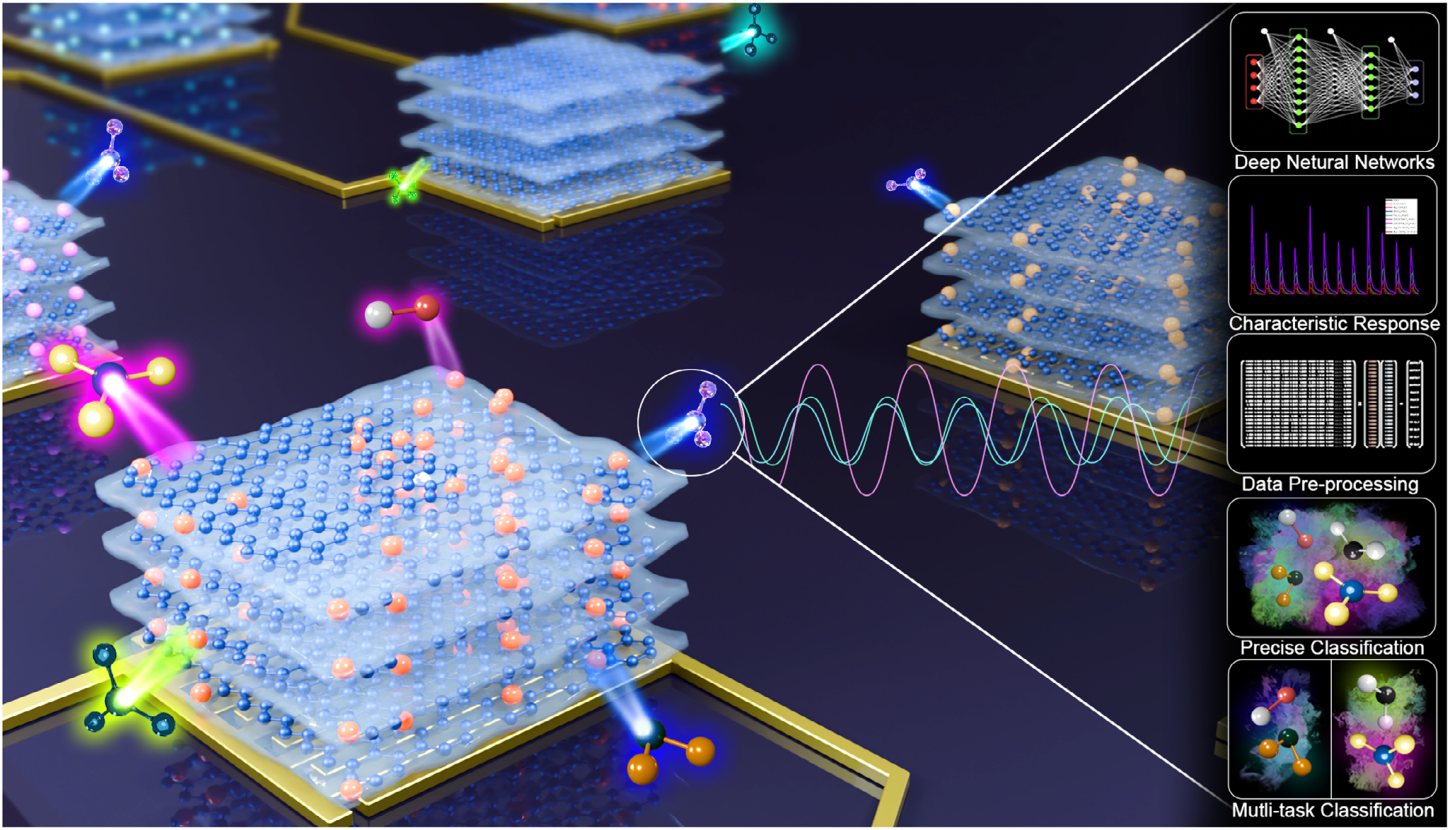
Schematic illustration of the simultaneous detection of multiple low‐concentration gases using a sensor array integrated with deep learning algorithms. The sensor array generates distinct characteristic responses to different gas molecules, which undergo data pre‐processing and are subsequently analyzed by deep neural networks. This integration enables precise classification and multi‐task learning for accurate gas identification and quantification.

**Figure** **1** illustrates how the sensor array responds concurrently to various gas molecules. Trace amounts of harmful gases present in everyday environments pose a substantial yet often overlooked threat to human health, making early detection imperative. To address this issue, various trace gases typically encountered in urban and industrial environments were developed, and the proposed rGO/tMO sensor array was utilized for precise identification and classification. Different concentrations of NO, NO₂, CO, and C₂H₅OH were introduced to the sensor array, observing distinct signal outputs corresponding to each gas. These signals were subsequently processed and analyzed using a deep learning framework, enabling accurate multitask classification of gas compositions and concentrations. To further validate the high‐precision classification capabilities of the system, simulations involving complex vehicle exhaust components, including mixtures of hydrocarbons and nitrogen oxides, were conducted. The sensor array effectively differentiated between various exhaust gas constituents and accurately identified different types of vehicle emissions with an accuracy of 98.22%. These findings highlight the potential of the sensor for the precise monitoring of vehicle emissions, underscoring the robustness and versatility of our sensor array when integrated with a deep learning model in real‐world applications.

### 2.2 Manufacturing Process and Sensor Array Design

Graphene and its derivatives, particularly graphene oxide (GO) and reduced graphene oxide (rGO), have been widely studied as promising materials for sensor applications because of their large surface area and exceptional electrical conductivity. GO is synthesized by oxidizing graphite layers and introducing abundant oxygen‐containing functional groups, such as hydroxyl, carboxyl, and epoxy groups. These functional groups impart hydrophilicity and high chemical reactivity, enabling functionalization via electrostatic adsorption or chemical bonding with metal ions and other species. However, the high oxidation level of GO reduces its electrical conductivity, limiting its effectiveness in high‐sensitivity gas sensors.

To improve the conductivity of GO, it is typically reduced to rGO using chemical agents (e.g., hydrazine and ascorbic acid) or thermal treatment, which partially restores its conjugated carbon structure by removing the oxygen functional groups. rGO, with its enhanced conductivity and remaining oxygen moieties, is well suited for functionalization, facilitating uniform metal oxide (MO) deposition. Although rGO itself possesses excellent electrical properties, integrating MOs into the rGO matrix substantially enhances sensor performance, particularly in terms of selectivity, response time, and sensitivity. MOs act as active sites for adsorption and catalysis, facilitating chemical reactions or electron transfer with the target gas molecules, thus amplifying the sensor response. The composite structure leverages rGO's high conductivity for improved electron transport, whereas the MOs provide chemical stability and gas‐specific interactions, enhancing sensitivity and stability.

rGO was chosen as the primary material for its superior conductivity and chemical stability. By doping with metal ions, creating carbon vacancies through high‐temperature treatment, and simultaneously forming metal oxides, the gas‐sensing performance of rGO is substantially enhanced compared to that of GO. This results in a composite material that retains the favorable properties of rGO while harnessing the catalytic and adsorption capabilities of the metal oxides, thus substantially improving sensor performance. By incorporating rGO, the limitations of traditional MO sensors that require high temperatures to operate were overcome, enabling an effective gas response at room temperature or lower. Each channel in the sensor array demonstrated unique sensing characteristics, enhancing selectivity across different gases.

**Figure 2:**
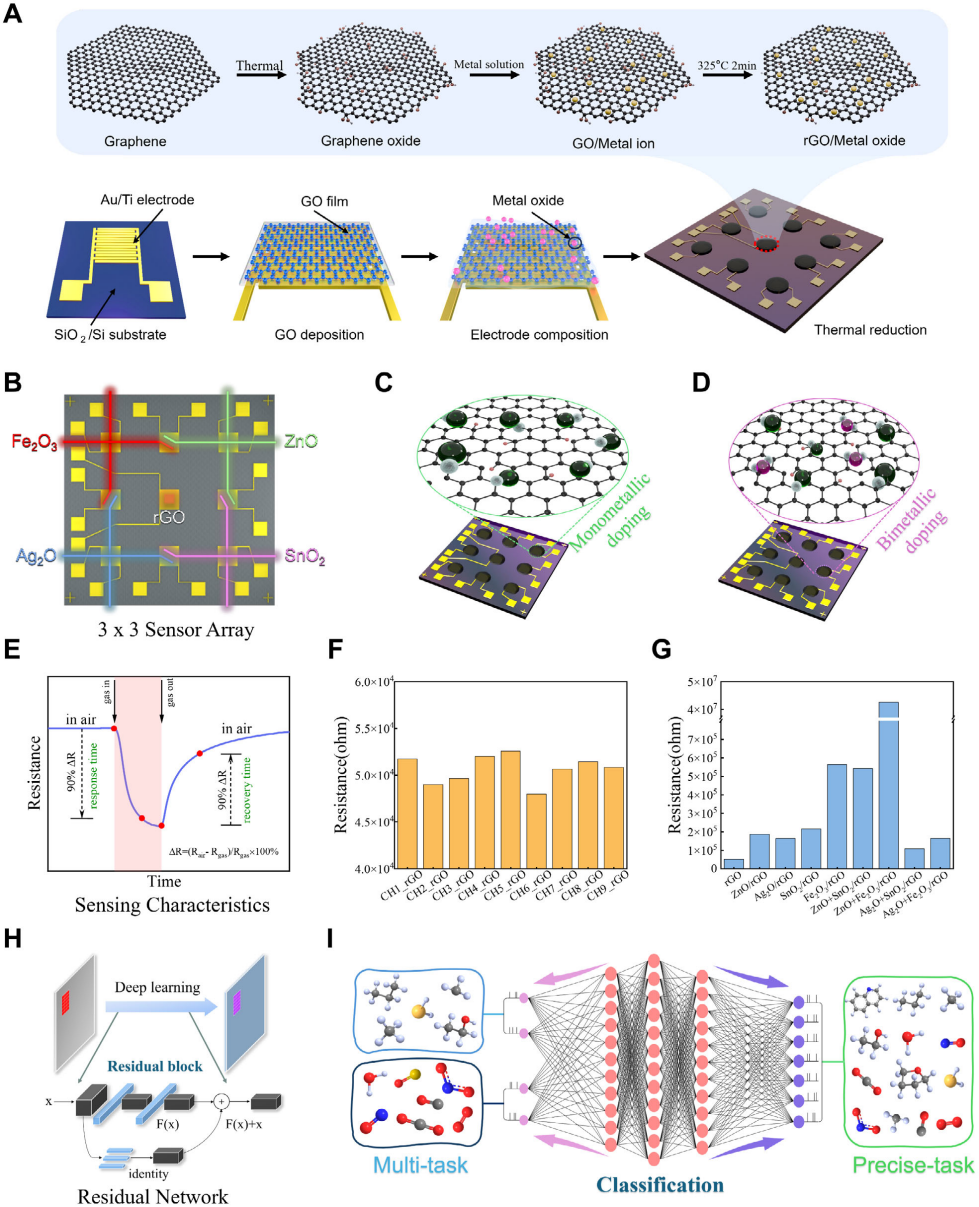
Schematic illustration of the fabrication and application of rGO‐based gas sensor arrays. A) Schematic depiction of the synthesis process for rGO/tMO, encompassing the thermal oxidation of graphene, metal‐ion coordination, and subsequent thermal reduction at 325 °C for 2 min. B) Final 3 × 3 nanosensor array structure. C) Schematic of rGO doped with single MOs. D) Schematic of rGO doped with binary MOs. E) Resistance response curves of the sensor exposed to target gases. F) Initial resistance values of the 9‐channel sensor array before doping. G) Resistance values of the 9‐channel sensor array after doping. H) Deep learning framework using residual blocks to process sensor signals. I) Multi‐task classification and precise‐task classification gas detection.

The synthesis process (**Figure** **2**A; Figure S1, Supporting Information) begins with the preparation of solutions of transition metals (Ag, Zn, and Fe) and a post‐transition metal (Sn), which are mixed with a graphene oxide solution. The metal ions were adsorbed onto the GO surface, and the mixture was deposited onto the electrodes via drop casting. The material then underwent vacuum drying and high‐temperature reduction at 325 °C to convert GO into rGO. This step improves adhesion and disperses a metal nanolayer uniformly across the rGO surface, forming MOs (Fe₂O₃, Ag₂O, ZnO, SnO₂) and ensuring a fine and homogeneous distribution within the rGO matrix. Furthermore, equimolar bimetallic oxide combinations (Fe₂O₃ + Ag₂O, Fe₂O₃ + ZnO, Ag₂O + SnO₂, ZnO + SnO₂) were synthesized and uniformly deposited on rGO using the same process. This culminated in the formation of a 3 × 3 sensor array (Figure 2B). Figure 2C,D illustrate the two types of MO‐functionalized rGO developed in this study: (c) rGO doped with a single MO (ZnO), and (d) rGO doped with bimetallic oxides (SnO₂). Figure 2E presents the sensing characteristics of the gas response along with the corresponding response formula. Figure 2F shows the resistance values of the nine original rGO channels before MO doping, and Figure 2G shows the changes in the resistance values for each channel after metal doping. These composites ensured precise MO distribution and strong adhesion between the sensor material and electrodes, leading to enhanced performance and stability. This method simplifies the fabrication process and guarantees high sensor stability and strong electrode adhesion. A substantial advantage of the proposed rGO/MO sensor arrays is their excellent performance at low operating temperatures. Unlike conventional gas sensors that often require elevated temperatures (≈200–300 °C) to achieve optimal sensitivity, these sensors operate effectively at temperatures below 20  °C. This low‐temperature operation substantially reduces energy consumption and enhances the safety and longevity of the sensors, making them ideal for practical applications, especially in scenarios requiring continuous, long‐term monitoring. Moreover, the intrinsic properties of the rGO/MO composites allow them to respond effectively without relying on external heating elements, thereby improving the overall efficiency and reliability of the sensing system. Notwithstanding these advances, practical research on the discrimination and analysis of multiple gases remains constrained, particularly under low‐concentration conditions due to a paucity of gas detection data. To address this challenge, a ResNet deep learning model with a distinctive residual structure was integrated for gas discrimination (Figure 2H). In the experimental setup, both precise‐task classification and multi‐task classification methods were employed (Figure 2I). The precise‐task classification employs a conventional single label for the identification and recognition of specific gases, whereas the multi‐task classification employs a multi‐label, multi‐task approach for the simultaneous classification of gas types and concentrations. This strategy not only improves the accuracy of the classification process but also reduces the complexity of the model, thereby increasing efficiency. The model benefits from shared representations by jointly learning multiple related tasks, which in turn leads to improved generalization and performance.

For the sensor array design, standard semiconductor wafer fabrication processes were employed using SiO₂ (1 µm)/Si (500 µm) wafers as substrates (Figure S1, Supporting Information). Photolithography was used to define and develop electrode patterns in the photoresist, followed by sputter deposition of a 10 nm titanium (Ti) adhesion layer and a 50 nm gold (Au) electrode layer. A lift‐off process was used to remove the excess photoresist and metal, forming precise Ti/Au electrode patterns. The wafer substrate was diced into individual chips to complete the fabrication process. The proposed sensor array comprises nine independent channels, each employing distinct sensing materials, which enable simultaneous real‐time detection across all channels.

Systematic characterization techniques confirmed that the designed rGO/MO sensor arrays were successfully deposited at the predetermined locations and effectively functionalized. Figure S2, Supporting Information shows the scanning electron microscopy (SEM) images of the nine sensing channels. The top‐view SEM images show that the MO nanoparticles were uniformly distributed on the rGO surface, indicating successful deposition and functionalization. Raman spectroscopy analysis further confirmed the effectiveness of the MO functionalization (Figure S3, Supporting Information). The MO‐functionalized rGO exhibited substantial changes in the D‐ and G‐band peaks compared with pristine rGO. Although these changes were relatively subtle, they were sufficient to demonstrate that even at a low doping level of 1%, the MOs successfully modified the surface structure of rGO. X‐ray diffraction (XRD) analysis (Figure S4, Supporting Information) was performed to further confirm these structural changes. Compared with pristine rGO, the MO‐doped rGO samples showed substantial diffraction peak shifts to lower angles. These shifts indicate crystal structure changes owing to successful MO doping, further confirming the effective incorporation of MOs (specific peak positions are detailed in Figure S4, Supporting Information).

### 2.3 Gas Sensing Properties

**Figure 3:**
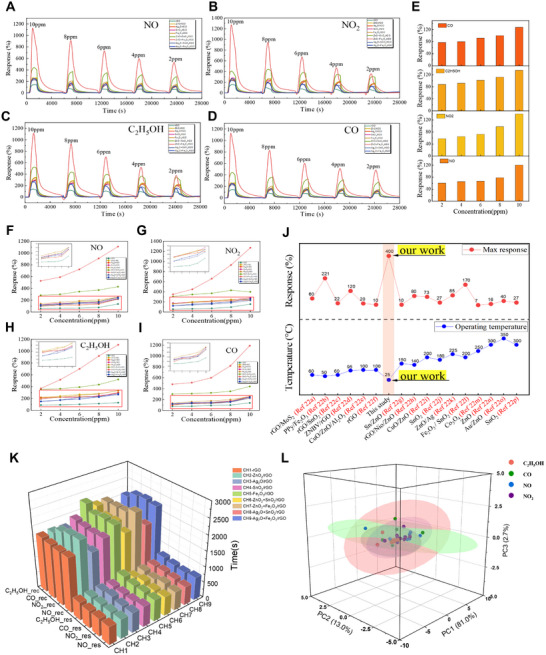
Investigation of the gas sensing characteristics of the rGO/tMO sensor array for detecting various harmful gases. A–D) Sensitivity of the two types of metal oxide (MO)‐doped reduced graphene oxide (rGO) sensors—one doped with a single MO and the other doped with a 1:1 mixture of MOs—toward four target gases: NO, NO₂, C₂H_5_OH, and CO, at concentrations of 2, 4, 6, 8, and 10 ppm. (E) Sensitivity of the original rGO material toward the four target gases (NO, NO₂, C₂H_5_OH, and CO). F–I) Maximum response sensitivity for each sensing channel under exposure to the four target gases. J) Comparison of the performance (maximum response and operating temperature) with similar sensing materials. K) Response and recovery times of each sensor material for the four target gases. L) Principal component analysis(PCA) result based on the sensitivity values of all channels of the optimized sensor array.

To verify the enhanced sensitivity of the proposed gas sensor array to various reducing and oxidizing gases, four target gases: nitric oxide (NO), nitrogen dioxide (NO₂), ethanol (C₂H₅OH), and carbon monoxide (CO) were tested at a low temperature of 20  °C. The total gas flow rate was maintained at 1000 sccm(standard cubic centimeters per minute), and a constant voltage of 1 V was applied. The response (R) was calculated as

(1) $$R=\left|\;\frac{R_{air}-R_{gas}}{R_{gas}}\;\right|\;\times 100\%$$

where *R*_gas_ represents the sensor resistance in the presence of the target gas, and *R*_air_ is the baseline resistance in air. Initially, the sensitivity of the original rGO without doping was measured, which was reduced solely by high‐temperature treatment, for each gas at a concentration of 10 ppm. The sensitivities of the undoped rGO were comparable across gases, ranging from ≈60%–140% (**Figure** **3**E). In contrast, the sensors in the array incorporating MO doping exhibited substantially higher sensitivity than pristine rGO. For instance, the SnO₂/rGO sensor showed an ≈100% increase in sensitivity, while the equimolar mixed MO system (SnO₂ + ZnO/rGO) demonstrated an improvement of about 140%. Notably, the Fe₂O₃ + ZnO/rGO sensor exhibited a nearly 1000% enhancement in performance (Figure 3A–D). An enhanced sensitivity was consistently observed across a range of concentrations (2 , 4 , 6 , 8 , and 10 ppm). Figure 3F–I presents the maximum response sensitivities for the target gases at different concentrations in each sensor array channel. The results indicated that rGO doped with ZnO and its mixed MOs demonstrated high response sensitivity across all concentrations of these gases. For example, the ZnO/rGO sensor achieved a maximum response sensitivity of ≈200% for 10 ppm NO₂, while the SnO₂ + ZnO/rGO sensor improved about 220% at the same concentration. The Fe₂O₃ + ZnO/rGO sensor achieved nearly a 1000% sensitivity enhancement for ethanol at 10 ppm compared to undoped rGO. In contrast, Ag₂O and its mixed oxide‐doped rGO showed relatively lower response sensitivities across all target gases, such as ≈150% for CO at 10 ppm. Overall, the rGO sensors doped with bimetallic oxides outperformed those doped with a single MO. Compared to undoped rGO, all doped rGO sensors exhibited substantial improvements in sensitivity for all target gases, with increases ranging from at least 100% to 200%.

The maximum response and operating temperature of the proposed gas sensor array were compared with those of several similar technologies that utilize MOs or GO (Figure 3J and Table S1, Supporting Information).^[^ ^22^ ^]^ Compared to traditional rGO sensors, the developed sensors demonstrated enhanced sensitivity of ≈200%, particularly in low‐concentration gas detection scenarios. This is largely attributed to optimized preparation methods and the integration of tMOs, which provide superior adsorption capabilities and electron transport. For example, in the work by Neeru Sharma,^[^ ^22f^ ^]^ rGO exhibited only a 10% response for specific selectivity to 10 ppm NO; in Yong Zhou's^[^ ^22a^ ^]^ work, the response was only 60% for 2 ppm NO₂ using rGO/MoS₂. However, the sensors doped with MOs typically require high operating temperatures. For instance, G. Neri^[^ ^22o^ ^]^ work reported that Au/ZnO showed only a 40% response for 100 ppm CO at an operating temperature of 350  °C, while Abeer Alhadi's^[^ ^22j^ ^]^ work demonstrated that Ag/ZnO showed only a 27% response for 50 ppm C₂H₅OH at an operating temperature of 300  °C. Unlike traditional MO‐based gas sensors, which typically require an operational temperature of 300  °C or higher, the developed sensor is capable of stable operation at temperatures as low as 20  °C. This low‐temperature functionality substantially reduces the energy consumption while minimizing the risk of thermal degradation of sensitive components, thereby improving the overall system reliability. Moreover, the sensor array developed in this study achieved a substantially higher maximum response at low temperatures, offering notable advantages in terms of operational simplicity, energy efficiency, and sensitivity.

In addition to sensitivity, two other critical performance metrics response time and recovery time were evaluated. The sensor array exhibited performance comparable to that of undoped rGO, with response times ranging from ≈600 to 700 s and recovery times ranging from ≈1000 to 1200 s (Figure 3K). Although the incorporation of tMOs substantially enhances the sensitivity of rGO sensors, the high‐temperature reduction process leads to the loss of oxygen‐containing functional groups, reducing the number of available adsorption sites, and potentially causing gas adsorption saturation. Principal component analysis of the sensitivity values across all channels indicated that the sensitivities were comparable within a specific range (Figure 3L), consistent with experimental observations. Despite the enhanced performance of doped materials, the limited variation in sensitivity across different gas concentrations poses challenges in differentiating multiple gases. Traditional classification methods, including support vector machines.^[^ ^23^ ^]^ and random forests, are inadequate for accurate gas classification in this scenario. These methods rely on distinct feature differences; however, the classification accuracy is compromised when feature distributions overlap owing to similar sensor responses. To overcome this limitation, a specialized residual network (ResNet)^[^ ^24^ ^]^ model capable of deep learning was developed. This model accurately identifies and classifies gases by learning subtle features that traditional methods fail to capture.

### 2.4 1D‐ResNet of Deep Learning for Multi‐Gas Identification

The complexity and volume of data from different gases at various concentrations, combined with similar sensing characteristics, such as sensitivity, response, and recovery time, underscore the necessity for accurate feature extraction in gas sensing. This study employed a multilayer neural network to classify gas types and quantify their concentrations from complex sensing data with high precision. We extended the ResNet architecture, which is known to address the vanishing gradient problem in deep networks via residual (skip) connections, by optimizing it as a 1D ResNet (1D‐ResNet) for efficient extraction and processing of complex gas sensing data (Figure S9, Supporting Information).

To evaluate the electrical signals from the sensor array, a multitask classification experiment was designed, involving multiple distinct label columns, producing two results simultaneously: (a) gas type and (b) gas concentration. This experiment aimed to distinguish between different gas types and concentrations, validate the feasibility of the model, and demonstrate its high performance in gas classification. To assess the robustness and advantages of this multitask classification, it was compared with a traditional single‐label precise classification task that focused on distinguishing specific combinations of gas types and concentrations. The results showed that the multitask classification approach substantially outperformed the traditional classification in terms of accuracy and overall performance, particularly for detecting gases at low concentrations. This finding emphasizes the effectiveness of multitask classification in handling complex gas sensing scenarios.

**Figure 4:**
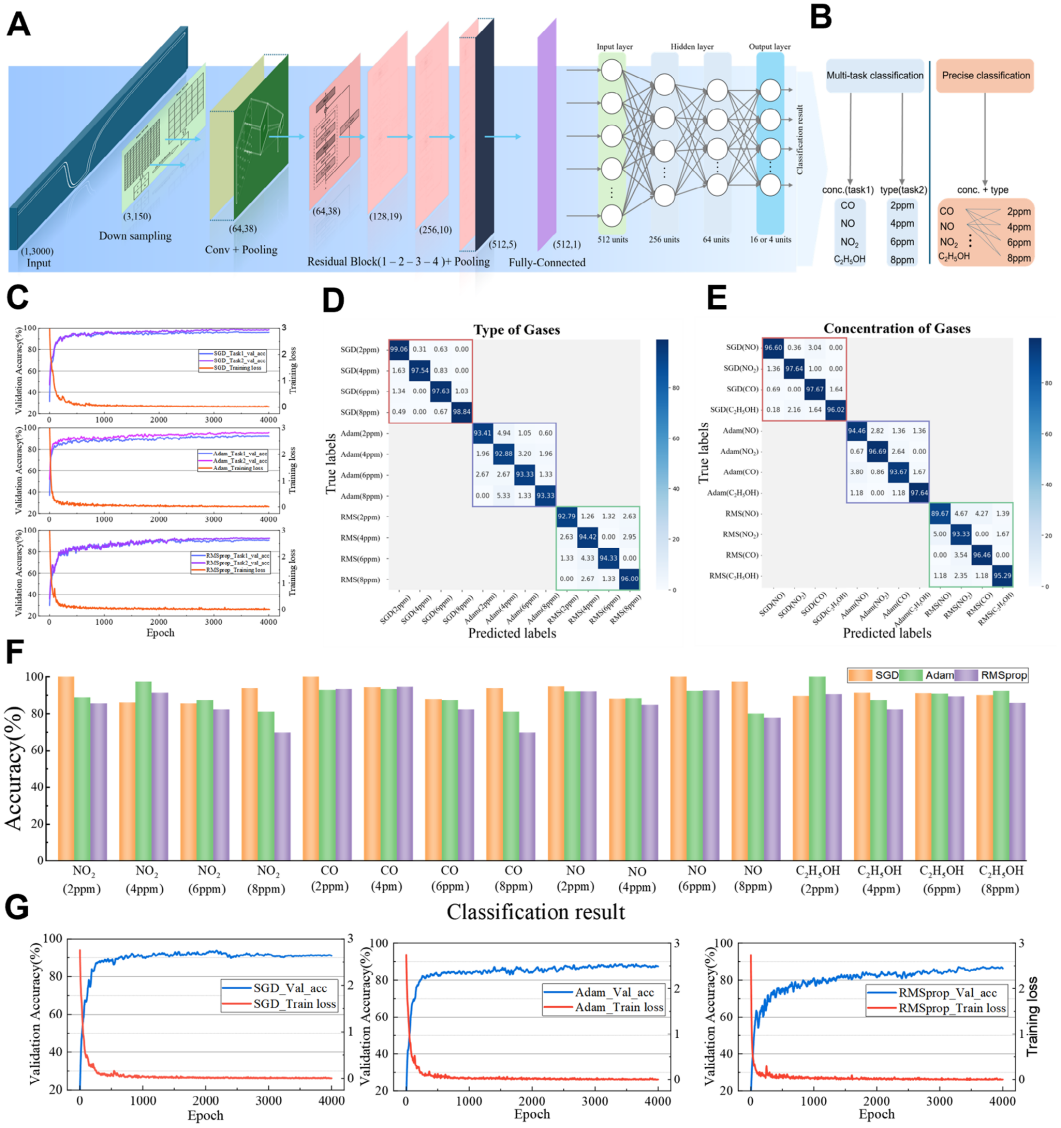
ResNet deep learning analysis process. A) Architecture of the 1D‐ResNet model used in the experiment, including the input layer, down sampling layers, convolutional layers, residual blocks, and fully connected layers. B) Structures for multi‐task and precise classification. The multi‐task classification encompasses tasks for identifying gas type and concentration simultaneously, while the traditional precise classification categorizes each gas at different concentrations as distinct classes. C) Accuracy and loss variations for multi‐task classification using different optimizers. The training and validation accuracy and loss curves for multi‐task classification using three different optimizers: SGD, Adam, and RMSprop. D) Confusion matrix for gas type classification (task 1) in multi‐task classification using three different optimizers. E) Confusion matrix for gas concentration classification (task 2) in multi‐task classification using three different optimizers. F) Comparison of highest classification accuracies achieved under three different optimizers for precise classification. G) Accuracy and loss variations for traditional precise classification using three different optimizers.

During data pre‐processing for training the 1D‐ResNet, raw data was collected from four gases—nitric oxide (NO), nitrogen dioxide (NO₂), ethanol (C₂H₅OH), and carbon monoxide (CO)—through repeated cycle experiments. The concentration of each gas (in ppm) was normalized against its maximum value to mitigate deviations owing to varying concentration ranges. For multitask classification, which was the primary experimental approach, 2016 samples (nine sensing channels × 14 cycle experiments × four concentrations × four gas types) were gathered. These samples were grouped and labeled according to gas type and concentration, as shown in **Figure** **4**B (left). For comparison, a traditional precise classification task was conducted using the same 2016 samples, with assigned specific labels for each combination of concentration and gas type, resulting in 16 distinct labels (4 gases × 4 concentrations), as depicted in Figure 4B (right).

Figure 4A presents a schematic of the ResNet model design. First, the preprocessed data were used as the input layer. The data were initially down sampling to reduce the volume, improve computational efficiency, and enhance the generalization ability of the model. Next, the data were processed via an average‐pooling layer to reduce dimensionality, followed by a convolutional layer, batch normalization, and a rectified linear unit activation function. The max‐pooling operation complements these steps. The data are then passed through four stages, each comprising multiple residual blocks, to perform the deep extraction of a feature. Subsequently, a global pooling structure was applied. The features from the global average pooling were fed into a fully connected layer for classification, and the results were visualized for analysis.

The training, validation, and test sets were allocated at a 70%:15%:15% ratio, respectively. All datasets were labeled with the corresponding real gas types and concentrations, enabling supervised learning. To ensure data precision and model reproducibility, a random seed was set to generate consistent random‐number sequences during dataset partitioning, model initialization, and training. This approach prevented potential overlap in dataset partitioning and enhanced the reliability of the experimental results.

To evaluate the effectiveness of our deep neural network for classification tasks, the impact of different optimizers on the model performance was examined. The optimizer is crucial in neural network training because it dictates the method and pace of weight updates, directly influencing both the convergence speed and the model's ultimate performance. Three standard optimizers were selected: stochastic gradient descent (SGD), adaptive moment estimation (Adam), and root mean square propagation (RMSprop). SGD is a classic optimization algorithm featuring a straightforward update method; however, it may suffer from slow convergence and a tendency to become trapped in local optima. Adam combines momentum and adaptive learning rate techniques, offering faster convergence and better global optimization capabilities. RMSprop was designed to address learning rate attenuation issues and is particularly effective for handling unbalanced data. Experiments were conducted to compare the training accuracy and the loss of the three optimizers across multi‐task and precise classification tasks.

For multitask classification, after optimizing the model parameters, the learning rate was set to 0.0004, and 4000 epochs were conducted. The experimental results showed that the validation set accuracy exceeded 90% for all three optimizers: SGD achieved 97.52% for Task 1 (gas type) and 96.85% for Task 2 (concentration), Adam achieved 94.5% for Task 1 and 92.5% for Task 2, and RMSprop achieved 90.5% for Task 1 and 91.5% for Task 2 (Figure 4C; Figure S11, Supporting Information). Notably, RMSprop exhibited substantial fluctuations during the first 2000 iterations but eventually converged monotonically to the target value. Among the three optimizers, SGD demonstrated the greatest adaptability, resulting in the best performance. The classification result for each optimizer was visualized using confusion matrices (Figure 4D,E), confirming that our ResNet model effectively distinguished the labeled categories under different conditions for each optimizer.

To further validate the superiority of our multi‐task approach, the performances of the three optimizers we evaluated in a precise classification task. The results showed accuracies of 92.5% for SGD, 90% for Adam, and 88% for RMSprop (Figure 4F; Figures S12–S14, Supporting Information). The loss and validation accuracies of the three optimizers were compared under identical parameter conditions (Figure 4G). The results highlighted the differences in accuracy, convergence speed, and stability during precise classification. Notably, multitask classification consistently achieved higher accuracy than precise classification across all optimizers, with the SGD optimizer demonstrating the best performance. This underscores the effectiveness of our multi‐task approach compared with traditional precise classification methods. Overall, the study findings indicate that the multitask classification approach provides a performance enhancement of ≈5% compared with the precise classification method across all optimizers. This improvement highlights the robustness and efficiency of our multitask model, particularly when using the SGD optimizer, for effectively handling complex classification tasks involving both gas type and concentration. The enhanced accuracy and stability demonstrated by the multi‐task framework make it a superior choice for applications that require precise and concurrent gas detection and classification.

### 2.5 Applied to Vehicle Exhaust Experiments

Building on the earlier demonstrations of the effectiveness of the 1D‐ResNet model in detecting and classifying low‐concentration gases within complex feature maps, the validation was extended by applying our sensor system and a deep learning model to the analysis of vehicle exhaust emissions, including simulated engine abnormalities. This approach demonstrated the potential applicability of the proposed method to real‐world environmental monitoring.

The proliferation of private vehicles has raised concerns regarding the harmful effects of vehicle exhaust on environmental pollution and human respiratory health. Governments worldwide are developing technologies to reduce emissions and implementing regulations to limit the release of pollutants into the atmosphere. Diesel exhaust contains substantial quantities of nitrogen oxides (NOx) and particulate matter, both of which are associated with respiratory diseases and carcinogenicity. In contrast, gasoline vehicle exhaust is rich in hydrocarbons and volatile organic compounds (VOCs) that contribute to air pollution phenomena, such as photochemical smog and acid rain. Figure S17, Supporting Information shows the exhaust emissions from diesel and gasoline engines. Establishing and enforcing rigorous standards to control vehicle emissions are crucial for mitigating air pollution and protecting public health. This can be achieved by accurately identifying engine types, differentiating various exhaust pollutants, detecting engine abnormalities through changes in exhaust composition, and implementing preventive measures to address environmental and health impacts.

**Figure 5:**
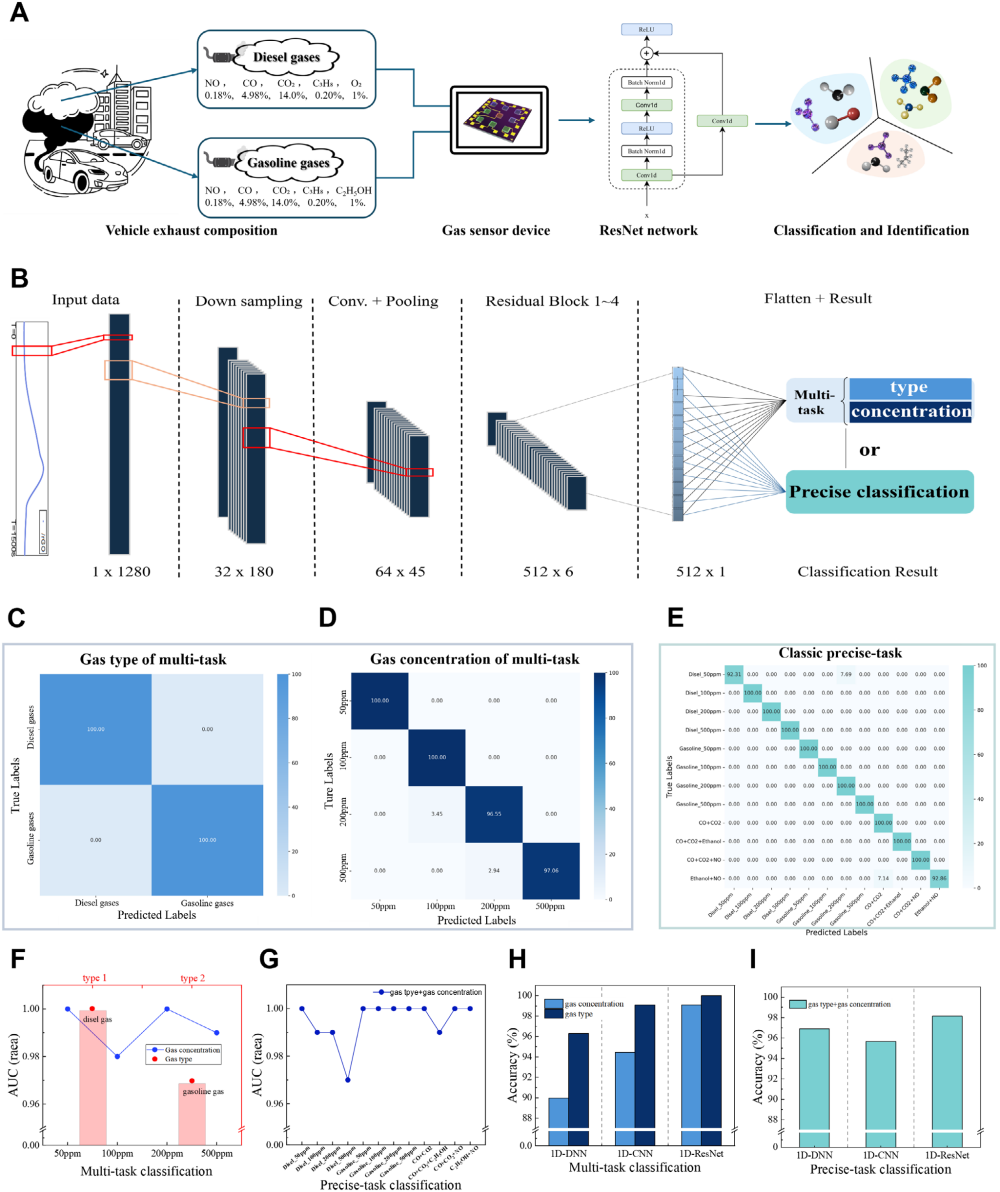
Gas generation process in the vehicle exhaust experiment and the 1D‐ResNet architectures utilized in this study. A) Generation process and composition of gases in vehicle exhaust. B) 1D residual network (1D‐ResNet) architecture. Accuracy results of multi‐task classification for C) gas type and D) gas concentration using 1D‐ResNet. I Accuracy results of precise‐task classification of 1D‐ResNet. AUC evaluation results for F) multi‐task classification and G) precise‐task classification. Performance comparison of 1D‐ResNet with other deep learning models (DNN and CNN) under H) multi‐task classification and I) precise‐task classification.

**Figure** **5**A illustrates the compositions of the diesel and gasoline vehicle exhaust gases and the experimental procedure for their analysis using deep learning. The exhaust gases used in our experiments contained components like NO, CO, CO₂, hydrocarbons (CH₄), C₂H₅OH, and O₂. These gases were processed using a gas sensor device, and the resulting data were analyzed using a ResNet network to classify the exhaust type, identify engine abnormalities, and determine the concentration of each component. Vehicle exhaust typically comprises a complex mixture of oxidizing and reducing gases, including hydrocarbons, NO_x_, and carbon oxides. The interactions between these gases complicate the analysis, making it challenging to apply the findings from studies focusing on single‐gas responses to these complex mixtures, especially at low concentrations. Moreover, analyzing trace quantities of individual gas components requires distinctive analytical methodologies for each gas, making comprehensive investigations both time‐consuming and costly. For example, NO_x_ detection typically employs infrared or ultraviolet absorption spectroscopy,^[^ ^25^ ^]^ while CO_x_ analysis often requires GC.^[^ ^26^ ^]^

**Figure 6:**
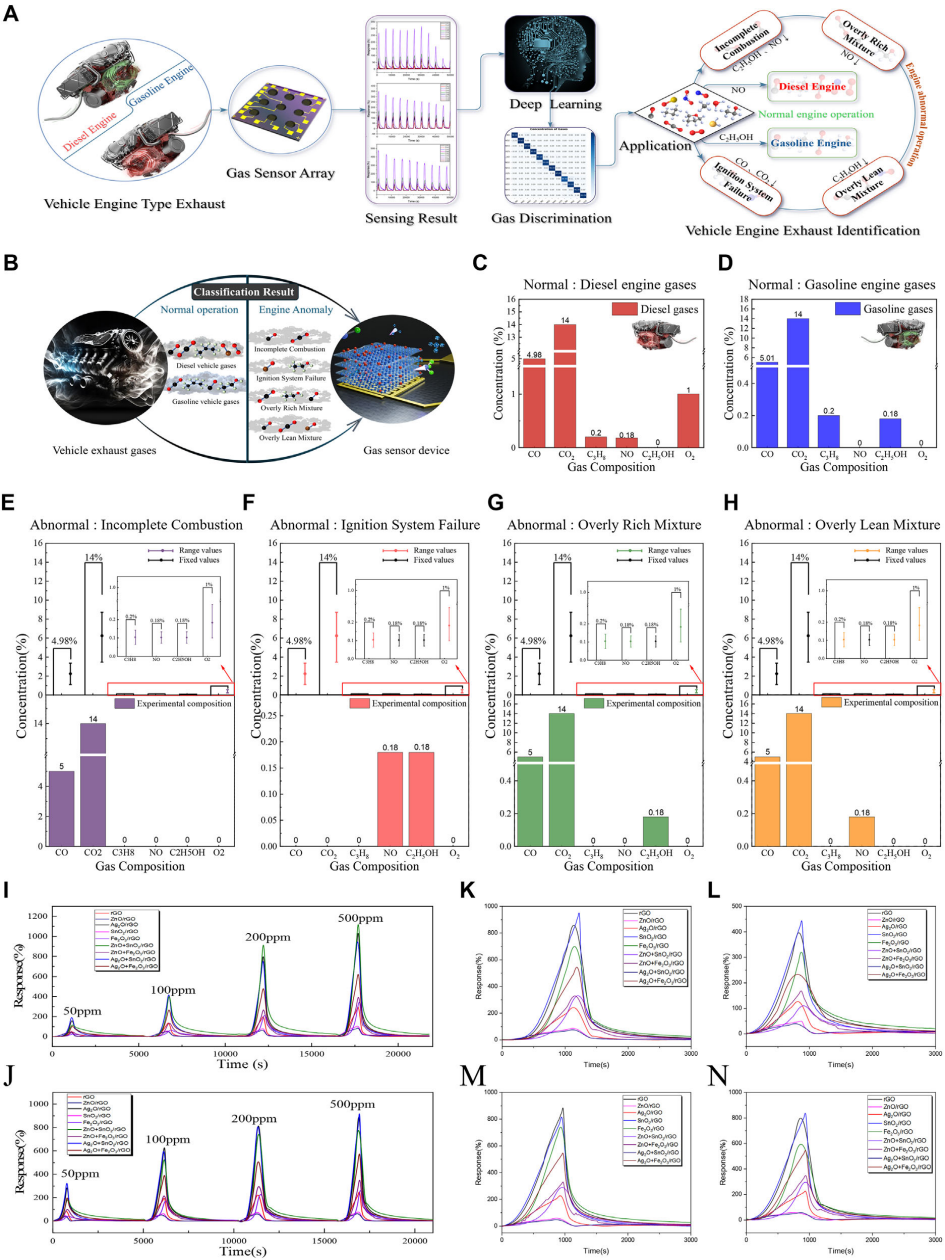
Gas sensor array workflow for exhaust gas discrimination and application in engine monitoring. A) Schematic of the integrated gas sensing system, from sensor array output to deep learning‐based gas discrimination and application in identifying exhaust emissions from gasoline and diesel engines. B) Classification results distinguishing normal and abnormal engine operations. Proportional compositions of gas molecules in vehicle exhaust under normal engine operating conditions: C) diesel exhaust gases and D) gasoline exhaust gases. Variations in the proportional compositions of gas molecules in vehicle exhaust under four typical engine fault conditions and the corresponding simulated gas compositions used in this study: E) incomplete combustion, F) ignition system failure, G) overly rich mixture, and H) overly lean mixture. Responses of the gas sensor array under varying concentrations (2.5%, 5%, 10%, and 25%) of exhaust gases: I) diesel exhaust gas comprising NO (0.18%), CO (4.98%), CO₂ (14.0%), C₃H₈ (0.20%), and O₂ (1%); J) gasoline exhaust gas comprising CO (5.01%), CO₂ (14.0%), C₃H₈ (0.20%), and C₂H₅OH (0.18%). Simulated responses of the vehicle under different fault conditions: K) incomplete combustion (CO + CO₂), L) ignition system failure (C₂H₅OH + NO), M) overly rich mixture (CO + CO₂ + C₂H₅OH), and N) overly lean mixture (CO + CO₂ + NO).

To address these challenges, experiments that simulated both normal and abnormal engine conditions were designed by introducing controlled variations in the exhaust gas compositions, and the experimental procedures were designed accordingly (**Figure** **6**A). Figure 6B displays all the simulated types of vehicle exhaust in this experiment, including diesel and gasoline exhaust under normal conditions and the four types under typical engine fault conditions. Exhaust gases were categorized based on the presence of NO and C₂H₅OH, varying by engine type and condition.^[^ ^27^ ^]^ Under normal conditions, gasoline exhaust contains CO, CO₂, C₃H₈, and C₂H₅OH, while diesel exhaust includes CO, CO₂, C₃H₈, O₂, and NO (Figure 6C,D; Figure S17, Supporting Information). Because numerous factors can cause variations in the exhaust gas concentrations or compositions, any deviation from the standard composition ratios can indicate potential engine faults. Establishing a precise standard to determine engine abnormalities is highly challenging; however, malfunctions are often reflected in the changes in the composition and concentration ratios of exhaust gases.^[^ ^28^ ^]^ Four representative experiments were designed to simulate changes in gas composition caused by engine faults: incomplete combustion,^[^ ^29^ ^]^ ignition system failure,^[^ ^30^ ^]^ overly rich mixture,^[^ ^27b^ ^]^ and overly lean mixture^[^ ^31^ ^]^ (Figure S18, Supporting Information). Figure 6E–H illustrates the range of changes in gas composition under each of the four typical engine fault conditions. Incomplete combustion, one of the typical failures, results in a relative increase in CO and CO₂ concentrations and a decrease in NO, C₂H₅OH, and hydrocarbons (C_x_H_y_) concentrations. This condition was simulated using exhaust gas containing only CO and CO₂ (Figure 6E). A mixture of NO and C₂H₅OH represented ignition system failure (Figure 6F). To simulate the overly lean mixture condition, a combination of CO, CO₂, and NO (Figure 6G) was used, whereas a mixture of CO, CO₂, and C₂H₅OH represented the overly rich mixture condition (Figure 6H). The rGO/tMO sensor array quantified the target gases and delineated their distinctive characteristics under normal and abnormal conditions. Consequently, 12 sets of experimental data were obtained, including simulations of the abnormal engine states (Figure 6I–N). This included four sets of response data under different low concentrations in the diesel engine exhaust (Figure 6I), four sets in the gasoline engine exhaust (Figure 6J), and experimental data simulating four typical engine faults (Figure 6K–N).

To adjust the 1D‐ResNet model and verify the specific responses of the sensor in actual vehicle exhaust tests, including abnormal conditions, the data were processed and segmented. Unlike the validation for a single gas, the varying lengths of the data responses necessitated treating the response of each channel as a complete feature set, each containing 1280 data points. Subsequent down sampling, convolution pooling, and processing with four residual blocks were performed. Finally, by simultaneously performing multiple classification tasks, a lightweight and more accurate multitask classification was designed for vehicle exhaust types and concentrations using multiple labels alongside a traditional precise classification task with a single label as a control (Figure 5B).

The results demonstrate that, even under simulated engine fault conditions, the proposed system achieves high‐precision classification and discrimination of vehicle exhaust gases. Specifically, using the proposed multitask classification model, 100% classification accuracy was achieved for the diesel and gasoline categories (Figure 5C), and 99.07% accuracy was obtained for various concentrations of the two exhaust types (Figure 5D). The traditional precise classification task achieved an accuracy of 98.7% (Figure 5E). Moreover, the testing time for the multi‐task classification required only 21.24 s compared to 39.39 s for traditional precise classification (Tables S2 and S3, Supporting Information), reducing the time by nearly 50%. Figure 5F,G present the AUC evaluation results for the multi‐task and precise classification models, respectively (Figures S19 and S20, Supporting Information). In the multitask classification (Figure 5F), the AUC values for different gas types (diesel and gasoline) and concentrations (50 , 100 , 200 , and 500 ppm) were consistently high, generally above 0.96. This indicates a strong discriminative ability to classify exhaust types and concentrations, even when engine abnormalities are present. For precise classification (Figure 5G), the model accurately classified each specific gas type and concentration combination, including those representing engine malfunctions, with AUC values typically close to 1.0, indicating high accuracy under normal and abnormal conditions. In addition, classification experiments were conducted on four typical simulated engine faults to evaluate the robustness of the proposed model in real‐world diagnostic scenarios. It achieved an accuracy of 98.22% when diagnosing these engine faults (Figure 5E). This result indicates that the designed sensor system is capable of efficiently recognizing exhaust gas types and performing outstanding discrimination in vehicle fault diagnosis. This capability is crucial for practical applications in vehicle diagnostics and emissions monitoring.

To further assess the overall feasibility of the experiment, two classic deep learning models, the DNN and CNN (Figure S10, Supporting Information), were developed based on the original 1D‐ResNet model, to serve as benchmarks. The classification accuracies of the DNN and CNN models were relatively low, attributed to the differences in adaptability and architectural complexity. Nonetheless, both models demonstrated robust performance. In the multitask classification, the DNN model achieved accuracies of 96.30% for type and 89.96% for concentration, whereas the CNN model achieved accuracies of 99.07% for type and 94.44% for concentration (Figure 5H; Table S2, Supporting Information). For precise classification, the DNN model achieved an accuracy of 97.53%, whereas the CNN model achieved an accuracy of 95.68% (Figure 5I; Table S3, Supporting Information). These results further substantiated the superior performance of the 1D‐ResNet model in handling complex classification tasks. Simulated vehicle exhaust experiments demonstrated that the sensor array exhibited high sensitivity to various gas mixtures, even at low concentrations, and effectively generated distinct characteristics. By leveraging these features, our deep learning model accurately identified gases within complex mixtures, extending beyond single‐gas identification. Consequently, our analysis achieved a high classification performance at low concentrations, substantiating the efficacy of the sensor array and deep learning system for practical engineering applications.

This study highlighted the substantial potential of integrating sensor technology with deep learning for precise gas monitoring and classification in complex environments. Given the limitations of the existing technologies in addressing the complexity and diversity of environmental issues, our approach offers a promising solution. We propose expanding the application of this technology to include real‐time monitoring, environmental sensing for autonomous vehicles, air quality management in smart cities, and the detection of hazardous gases during industrial accidents. In addition, the development of advanced sensor materials and systems and the establishment of a global gas profile database are essential to promote standardization and meet the complex demands of real‐world applications.

## 3 Conclusion

In this study, we developed a multichannel gas sensor array based on reduced graphene oxide (rGO) and trace metal oxides (tMOs), formed by doping with metal ions and employing high‐temperature reduction to introduce carbon vacancies. This design demonstrated enhanced sensitivity and selectivity for detecting low‐concentration gases. Furthermore, the potential of leveraging sensor signals as a foundation for deep learning‐based analysis and identification of complex gas compositions was comprehensively explored, demonstrating its promise as a diagnostic tool in challenging environments.

Our sensor system utilized nine independent sensor arrays, each functionalized with distinct sensing materials, to construct a comprehensive sensing platform. This design effectively mitigated critical challenges such as cross‐sensitivity and low selectivity. Functionalizing rGO with different tMO sensing materials on individual electrodes ensured the generation of unique electrical signals, reducing redundancy and enabling high‐response gas identification even at low concentrations. Additionally, the integration of rGO/tMO composites allowed the sensor array to operate efficiently at low temperatures (≈20 °C), overcoming the high‐temperature limitations of conventional metal oxide sensors. Notably, the rGO/tMO composites exhibited superior adsorption and catalytic properties, enhancing sensitivity and enabling sub‐ppm gas detection. For example, the Fe₂O₃ + ZnO/rGO sensor demonstrated nearly a 1000% increase in sensitivity to 10 ppm ethanol compared to pristine rGO (Figure 3H).

The achievement of this work lies in the application of artificial intelligence, particularly a multitask learning framework, to classify and identify different electrical signals derived from the sensors. By combining the processed experimental data with a 1D ResNet (1D‐ResNet) model, we successfully classified and recognized multiple gas types and concentrations simultaneously. To the best of our knowledge, this is the first demonstration of multi‐task classification in gas sensing, offering improved accuracy while reducing computational complexity compared to traditional methods. The multitask learning strategy leverages shared representations among related tasks, substantially enhancing model generalization and performance, particularly for complex scenarios such as automotive exhaust mixtures. These findings highlight the advantage of our approach in addressing the challenges of gas sensing in intricate environments.

Despite these accomplishments, several limitations remain. First, data processing and model training require substantial computational resources, which may hinder real‐time implementation in resource‐constrained settings. Second, the performance of the sensor array under highly variable environmental conditions, such as fluctuating humidity and temperature, warrants further investigation. Furthermore, although the fabrication process was optimized for uniformity and reproducibility, scaling up production to meet industrial standards remains a challenge. Future work should focus on developing miniaturized and portable systems to enhance usability. Integrating microchannel systems for gas flow control could further improve practical applicability.

In conclusion, this study presents an efficient sensing system capable of detecting and identifying complex gas mixtures. By integrating sensor arrays with deep learning models, it provides a robust platform for accurate gas recognition in complex environments, laying the foundation for the development of high‐precision, real‐time gas monitoring systems. This approach holds great promise for applications in environmental monitoring, vehicle emission control, and industrial safety.

## 4 Experimental Section

### Preparation of Pre‐MO/GO Solution by Solvent Blending Method

In this experimental section, four distinct solutions were prepared by dissolving the following salts in 70 µL of deionized water each: Fe (NO₃)₃·9H₂O(0.72464 mg), SnCl₂·2H₂O(0.19008 mg), AgNO₃(0.15748 mg), and Zn(NO₃)₂·6H₂O(0.456621 mg). These solutions were thoroughly mixed and stirred at 1500 rpm for 60 s. Each solution was then gradually combined with ten 100 µL aliquots of a 1 wt% aqueous GO solution, ensuring complete mixing after each addition, to reach a final volume of ≈1 mL. This process yielded four distinct 1 wt% metal ion/graphene oxide (M⁺/GO) mixtures, where M⁺ represents the respective metal ions: Fe^3^⁺, Sn^2^⁺, Ag⁺, and Zn^2^⁺. Each mixture underwent further processing steps, including 60 s of stirring, followed by 10 min of ultrasonication, and an additional 60 s of stirring. These steps resulted in pre‐treated 1 wt% Fe^3^⁺/GO, 1 wt% Zn^2^⁺/GO, 1 wt% Sn^2^⁺/GO, and 1 wt% Ag⁺/GO solutions.

For the mixed material solutions, to prepare 1:1 equimolar mixtures, half the mass of each metal salt was used: Fe(NO₃)₃·9H₂O(0.36232 mg) combined with Zn(NO₃)₂·6H₂O(0.2283 mg); Fe(NO₃)₃·9H₂O(0.36232 mg) mixed with AgNO₃(0.07874 mg);SnCl₂·2H₂O(0.09504 mg) paired with AgNO₃(0.07874 mg); Zn(NO₃)₂·6H₂O(0.2283 mg) joined with SnCl₂·2H₂O(0.09504 mg). Each pair of salts was dissolved in 70 µL of deionized water and subjected to the same processing steps as the individual solutions, ultimately yielding eight different precursor solutions.

### Preparation of the MO/rGO

A cleaned wafer was prepared, and 2 µL of each of eight different precursor solutions were deposited onto eight separate electrode surfaces. Additionally, one electrode was coated with 2 µL of a 1 wt% aqueous GO solution to serve as a reference. This procedure yielded a complete array of nine sensors. The array was subsequently placed in a vacuum chamber and left to dry for 20 h to ensure the complete evaporation of any surface moisture. Following the drying process, the samples were rapidly transferred to a furnace preheated to 325 °C and heated for 110 s. After heating, the samples were removed and allowed to cool in ambient air, thereby completing rGO/tMO sensor array fabrication.

### Sensor Device Measurements of Gas Sensing

The gas measurements were performed using specialized measurement and monitoring systems. A direct current (DC) bias voltage of 1 V was applied to all the sensing channels via internal probe tip arrays aligned with the interdigitated electrode patterns. The resistance of each sensing channel was simultaneously monitored using a Keithley 7001 switch system, which sequentially accessed each channel of the interface circuit at 1‐s intervals. Subsequently, a Keithley 2612A sourcemeter was used to measure the electrical response of the sensor array. The resistance values, serving as sensing signals, were recorded on a computer connected to an IEEE‐488 GPIB interface (Figure S15, Supporting Information). Target gases were introduced after the response of the sensor to dry air reached full saturation and the initial baseline resistance stabilized. Nitrogen was used as a baseline gas to detect the target gases. The gas flow was maintained at 1000 sccm.

### Data Pre‐Processing and 1D‐ResNet Deep Learning Framework

The complete dataset encompassed measurements from all the channels of the sensor array, covering 16 target gas conditions (four gas types × four different concentrations), various material compositions within the 3 × 3 sensor array, and 14 cycles of repeated experiments, resulting in 2016 training datasets. Each dataset comprised raw signal sequences containing 3000 data points. For data pre‐processing, a convolutional down‐sampling layer was initially applied, reducing the data dimensionality to the shape of (3, 150). The data then underwent two convolutional layers followed by pooling operations, resulting in an intermediate shape (64, 37). Subsequently, four residual blocks were employed with strides of 1, 2, 2, and 2, yielding the final data shapes of (512 and 5).

For classification, the processed data were fed into two independent, fully connected layers to produce the final classification outputs. Throughout the CNN, the convolutional layers utilized a kernel size of 7, stride of 2, and padding of 3. The network was trained using a batch size of 32 and a learning rate of 0.0004. The use of different optimization algorithms (SGD, Adam, and RMSprop) was explored in separate experiments to evaluate their effects on the model performance.

### Vehicle Exhaust Experiment

The exhaust gases used in this study were standard gases that were rigorously evaluated and certified by the Korean Laboratory Accreditation Scheme (KOLAS), operated by the National Institute of Technology and Standards under the Ministry of Trade, Industry, and Energy of the Republic of Korea. Detailed information on these gases is provided in Figure S16, Supporting Information. The concentrations of the two exhaust gases were diluted to 1/40, 1/20, 1/10, and 1/4 by mixing them with dry air to achieve a total gas flow rate of 1000 sccm, which was controlled using mass flow controllers. The exhaust gas mixtures were tested in dry air at room temperature with the concentration of the interfering gas set to 10 ppm during additional tests. The target gases were introduced after ensuring the complete saturation of the sensor response to dry air and stabilization of the initial baseline resistance. A total of 1080 datasets were collected (12 experimental conditions × 9 sensor arrays × 10 experimental cycles) using the same 1D‐ResNet framework with optimized parameters. In addition, two other classical deep learning models (DNN and CNN) were implemented for comparative analysis.

## Conflict of Interest

The authors declare no conflict of interest.

## Author Contributions

T.L. developed the concept and conceived the experiments. T.L. and Y.J.H. conducted the theoretical analyses and carried out the experiment. Y.J.H. and L.Z. provided technical support for the theory and experiment. T.L., J.H., and T.H. performed experimental data analyses. T.L. drafted the manuscript with support from all coauthors. S.C.J. revised and finalized the paper.

## Supporting information



Supporting Information

## Data Availability

Research data are not shared.
