# Supplementary material for: Hybrid Series of Carbon‐Vacancy Electrodes for Multi Chemical Vapors Diagnosis Using a Residual Multi‐Task Model
Source: Adv Sci (Weinh). 2025 May 11;12(25):2500412. doi: 10.1002/advs.202500412 (PMC12224976; doi:10.1002/advs.202500412)
Supplement: Supplementary file 1 — Supporting Information [file ADVS-12-2500412-s001.docx]

Supporting Information

**Hybrid series of carbon-vacancy electrodes for multi chemical vapors diagnosis using a residual multi-task model**

Tianci Liu, Yun Ji Hwang, Lu Zhang, Jongwoo Hong, Teajong Hwang, and Seong Chan Jun^*^


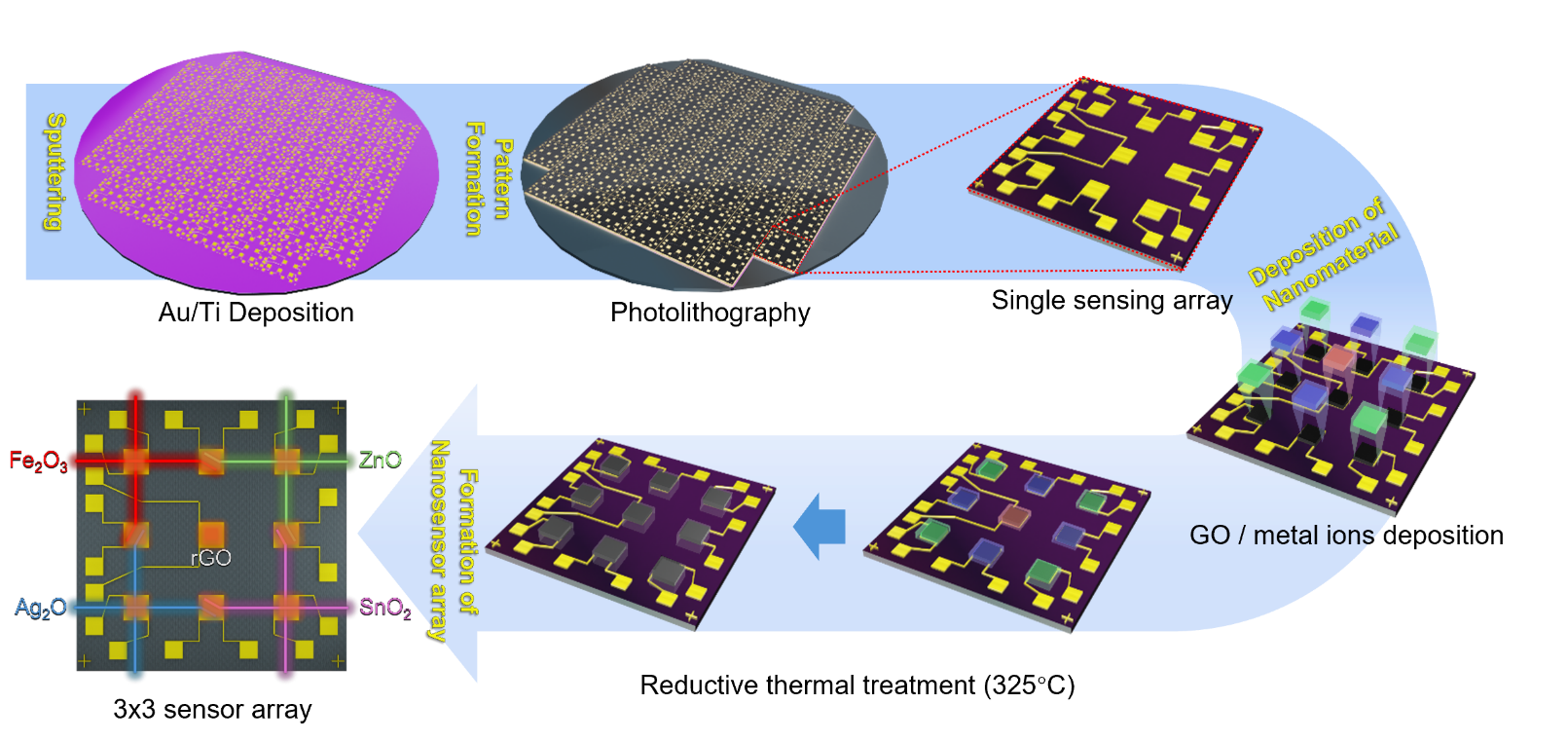


**FigureS1.** Gas sensor array synthesis process.


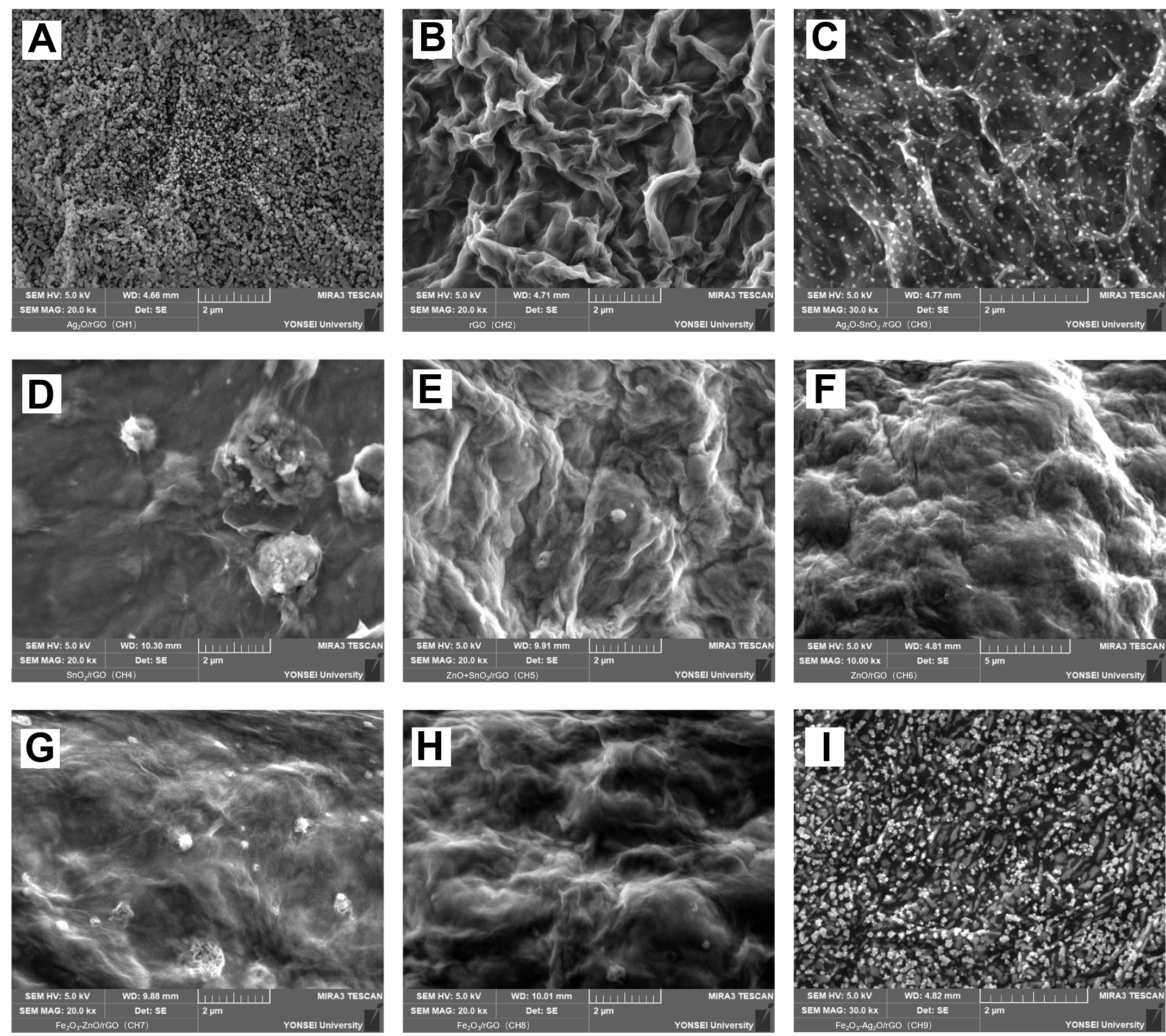


**FigureS2.** Top-view scanning electron microscopy (SEM) images of all sensing channels of the rGO/ tMO sensor array: (A) Ag_2_O/rGO, (B) rGO, (C) Ag_2_O-SnO_2_ /rGO, (D) SnO_2_/rGO, (E) ZnO+SnO_2_/rGO, (F) ZnO/rGO, (G) Fe_2_O_3_-ZnO/rGO, (H) Fe_2_O_3_/rGO, and (I) Fe_2_O_3_-Ag_2_O/rGO.


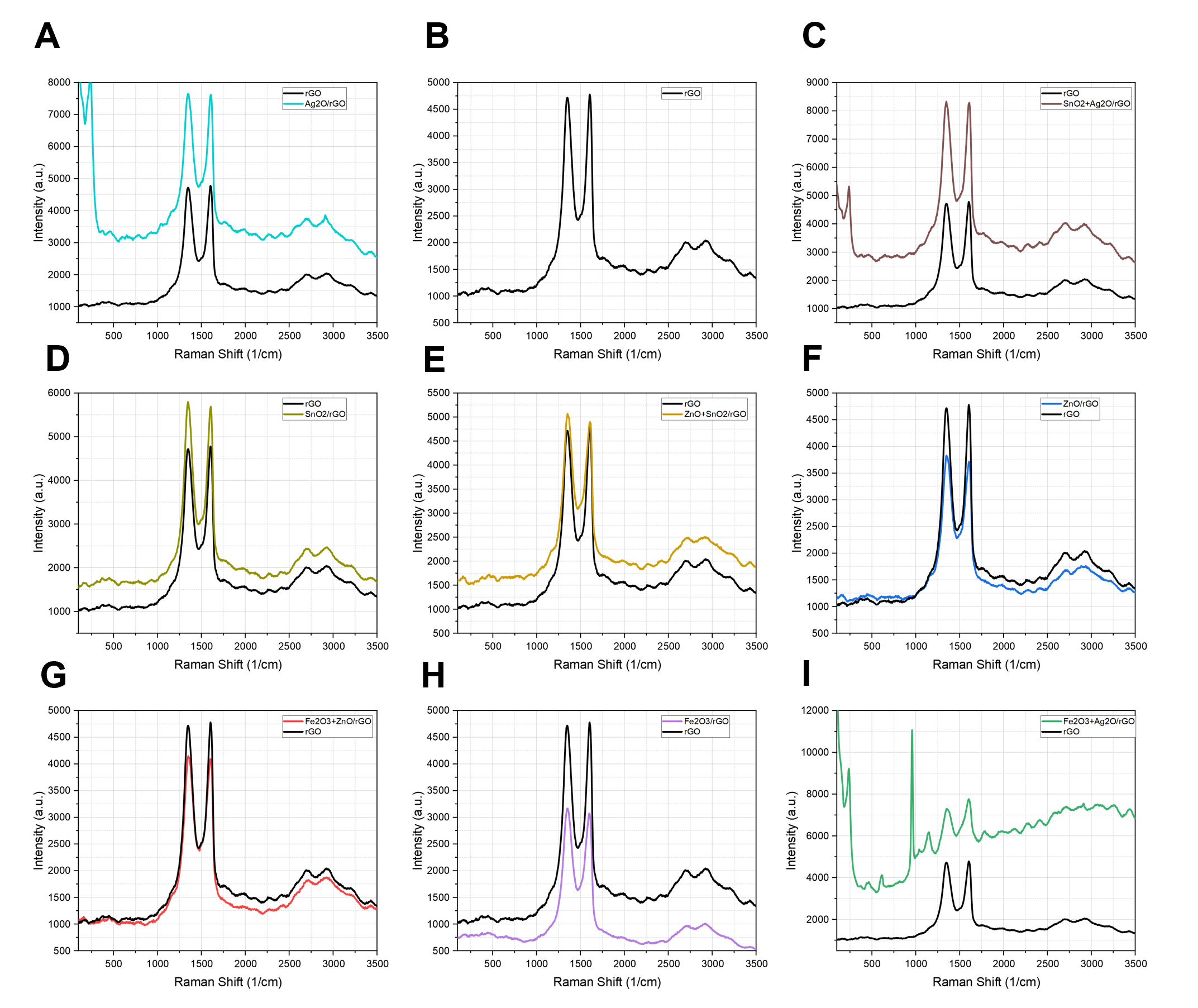


**FigureS3.** Raman spectroscopy images of the rGO/ tMO channels: (A) Ag_2_O/rGO, (B) rGO, (C) Ag_2_O-SnO_2_ /rGO, (D) SnO_2_/rGO, (E) ZnO+SnO_2_/rGO, (F) ZnO/rGO, (G) Fe_2_O_3_-ZnO/rGO, (H) Fe_2_O_3_/rGO, and (I) Fe_2_O_3_-Ag_2_O/rGO.


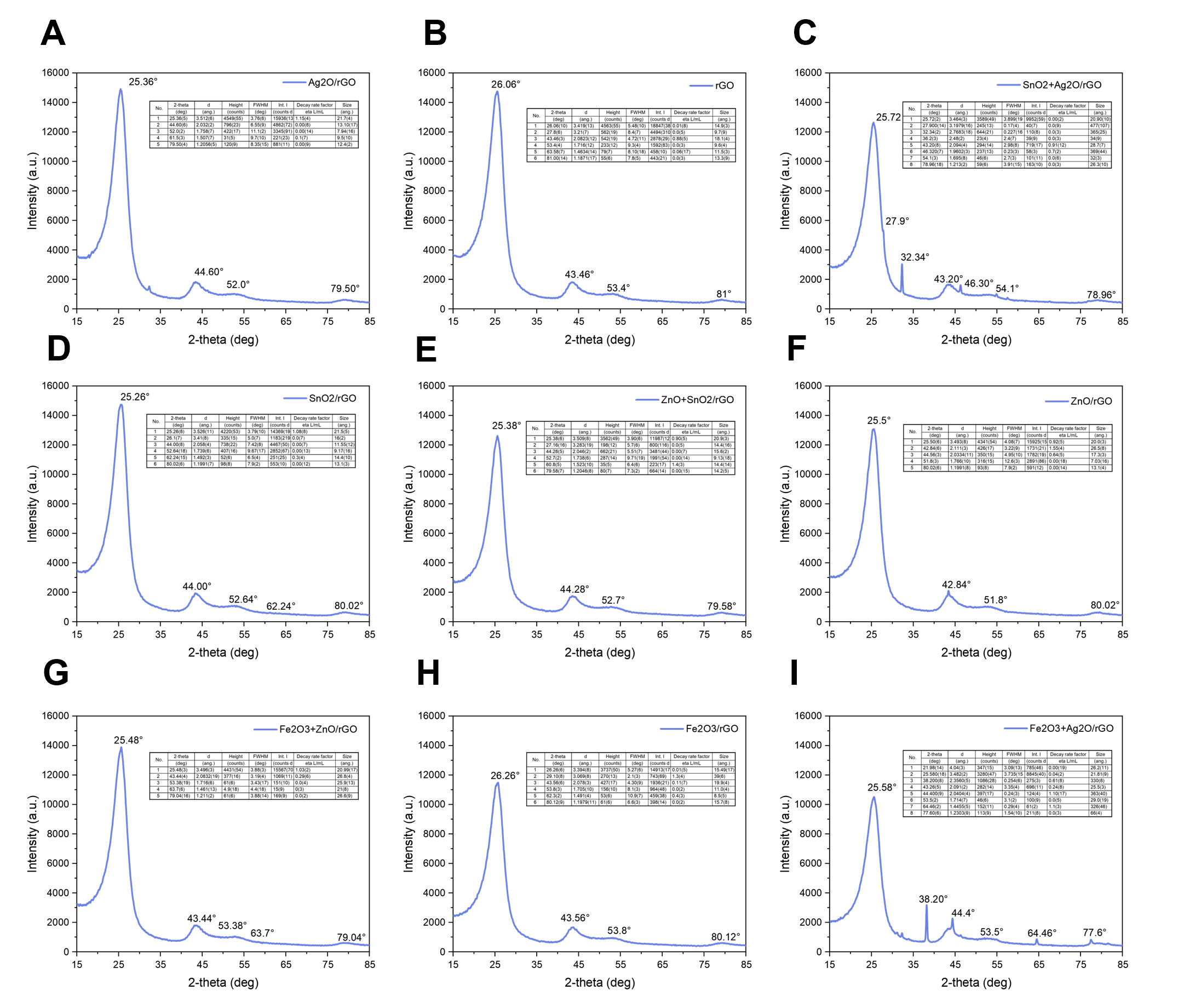


**FigureS4.** X-ray diffraction (XRD) patterns of all rGO/ tMO sensor array channels: (A) Ag_2_O/rGO, (B) rGO, (C) Ag_2_O-SnO_2_ /rGO, (D) SnO_2_/rGO, (E) ZnO+SnO_2_/rGO, (F) ZnO/rGO, (G) Fe_2_O_3_-ZnO/rGO, (H) Fe_2_O_3_/rGO, and (I) Fe_2_O_3_-Ag_2_O/rGO.


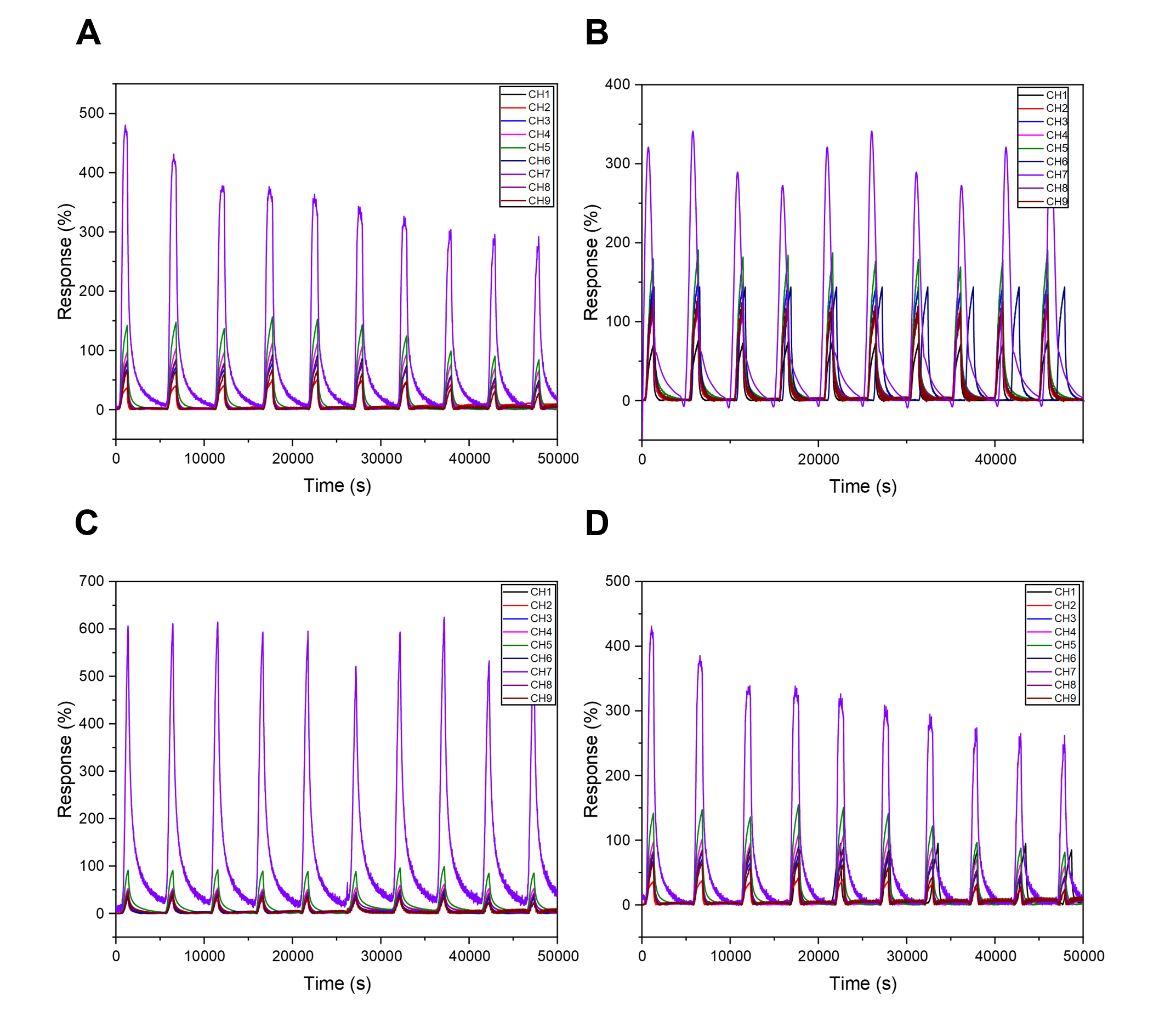


**FigureS5.** Response of the rGO/tMO sensor array at 2ppm concentration of: (A) NO_2_, (B) NO, (C) CO, and (D) C_2_H_5_OH.


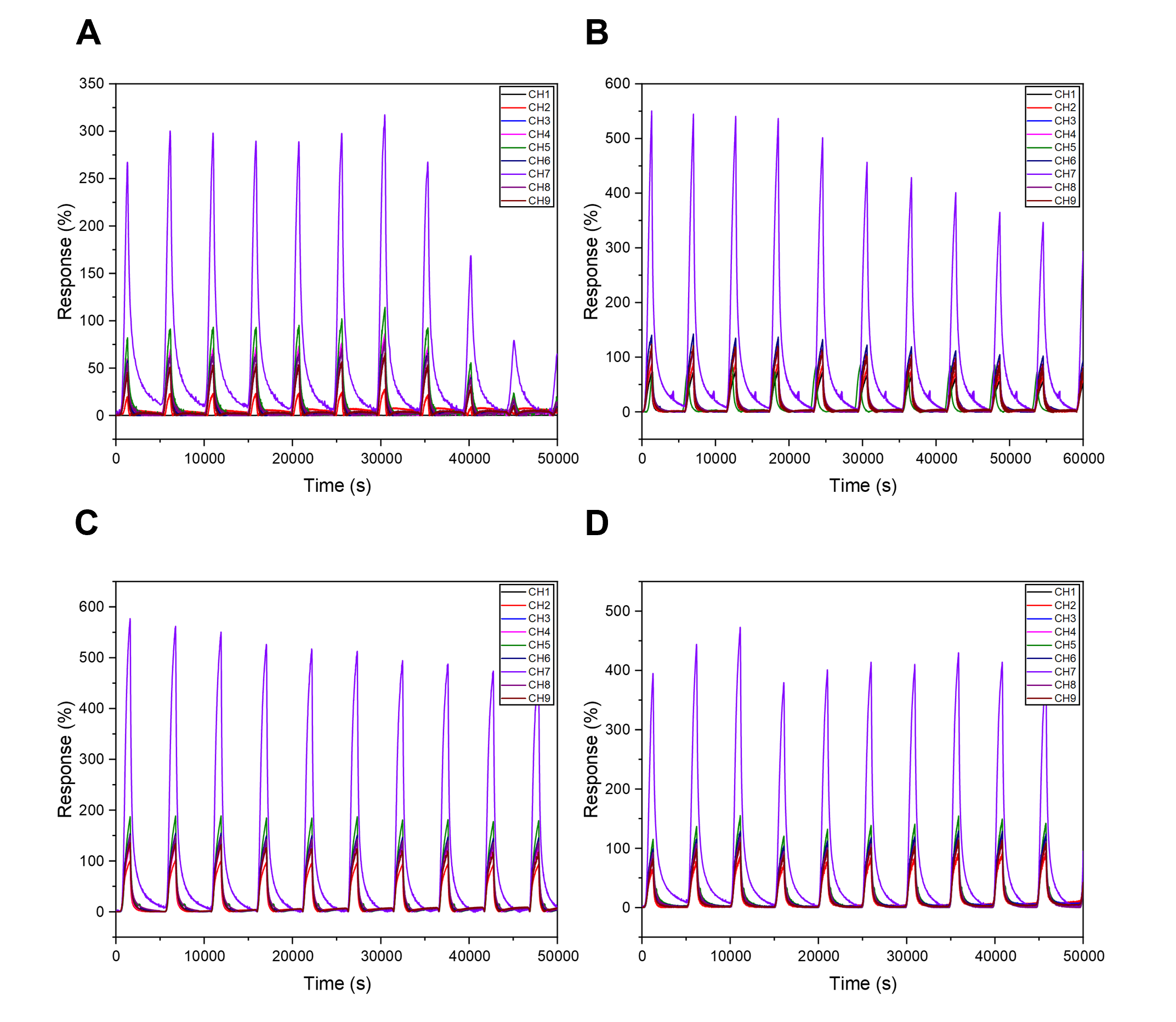


**FigureS6.** Response of the rGO/tMO sensor array at 4ppm concentration of: (A) NO_2_, (B) NO, (C) CO, and (D) C_2_H_5_OH.


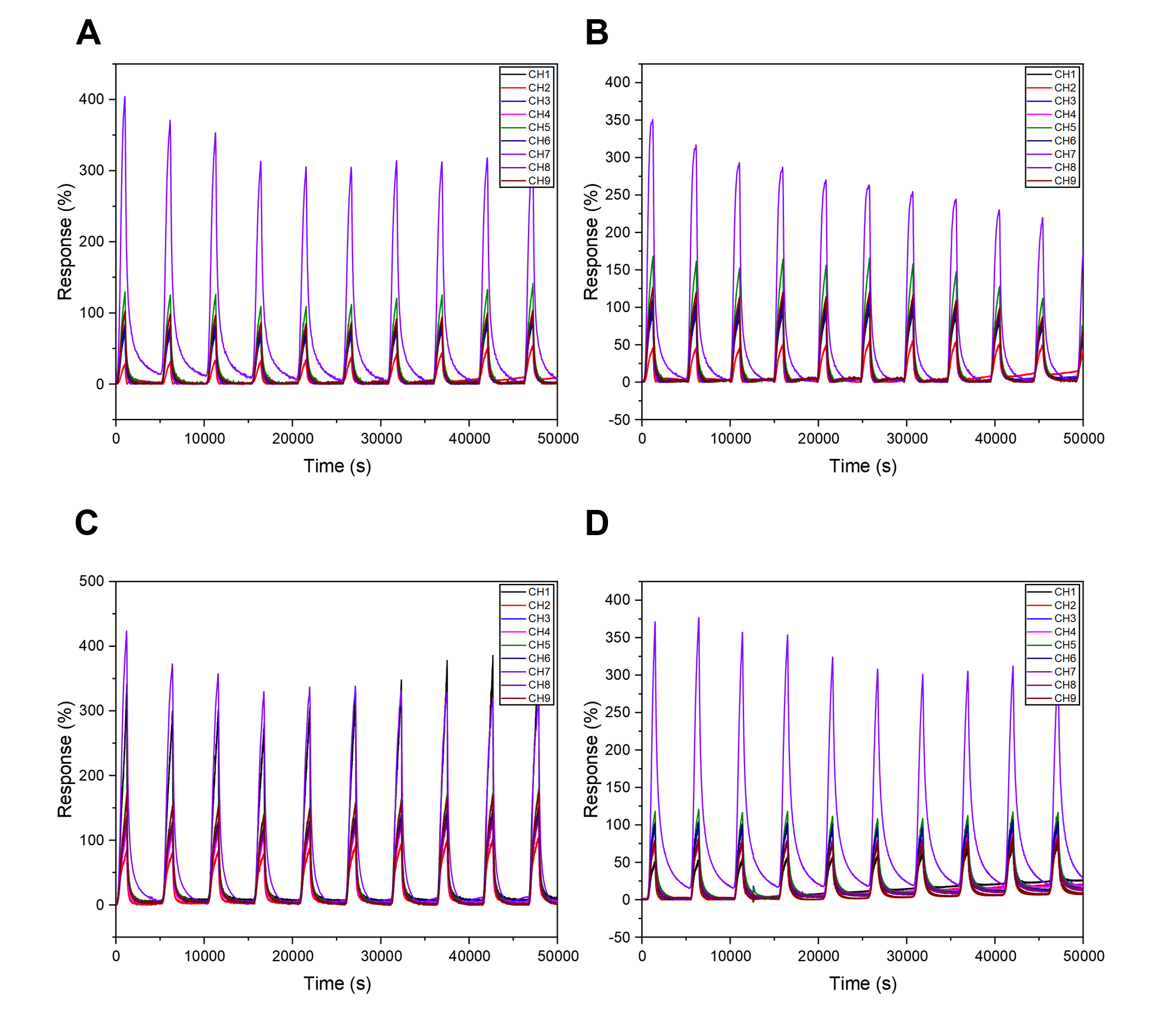


**FigureS7.** Response of the rGO/tMO sensor array at 6ppm concentration of: (A) NO_2_, (B) NO, (C) CO, and (D) C_2_H_5_OH.


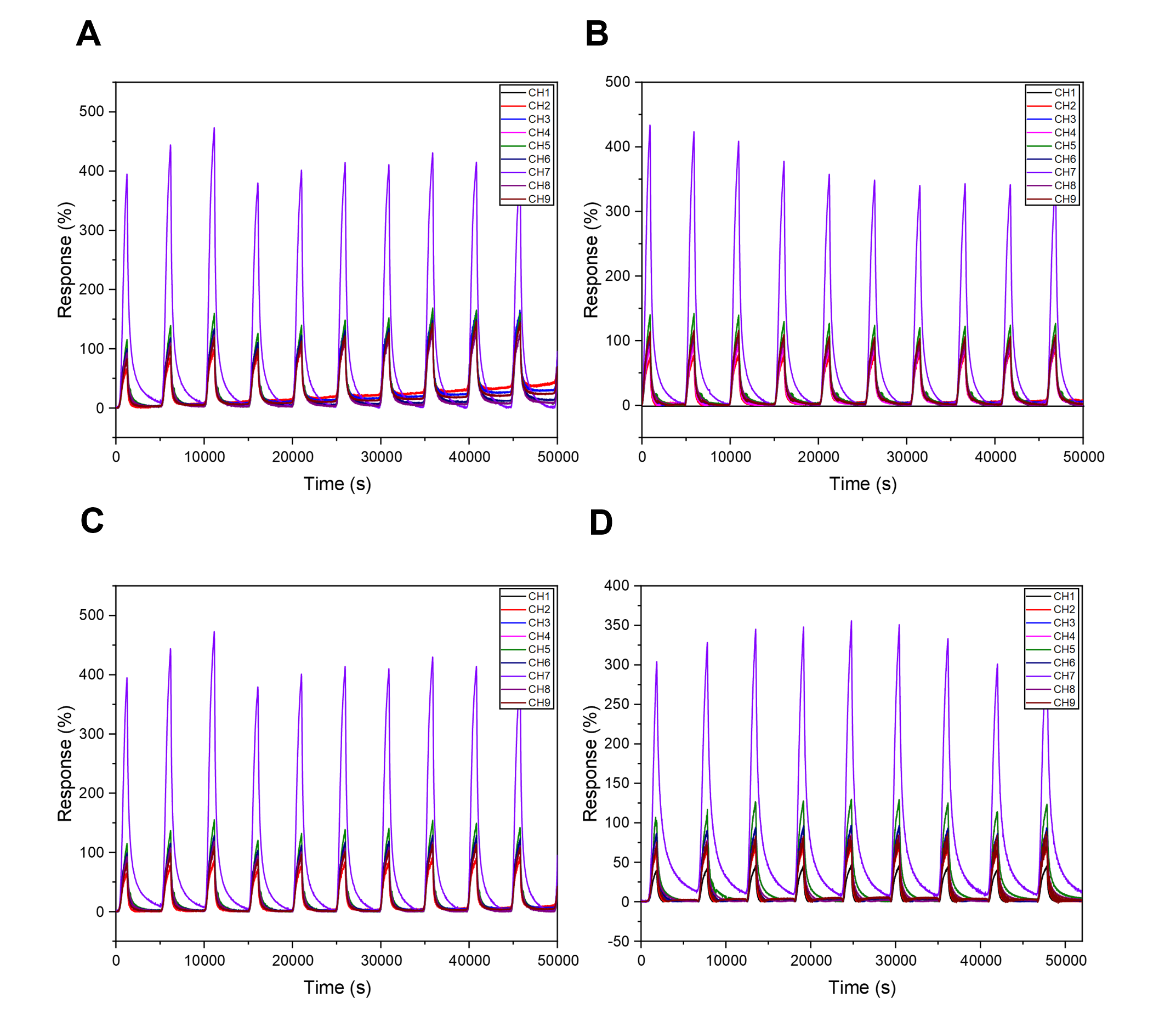


**FigureS8.** Response of the rGO/tMO sensor array at 8ppm concentration of: (A) NO_2_, (B) NO, (C) CO, and (D) C_2_H_5_OH.

**
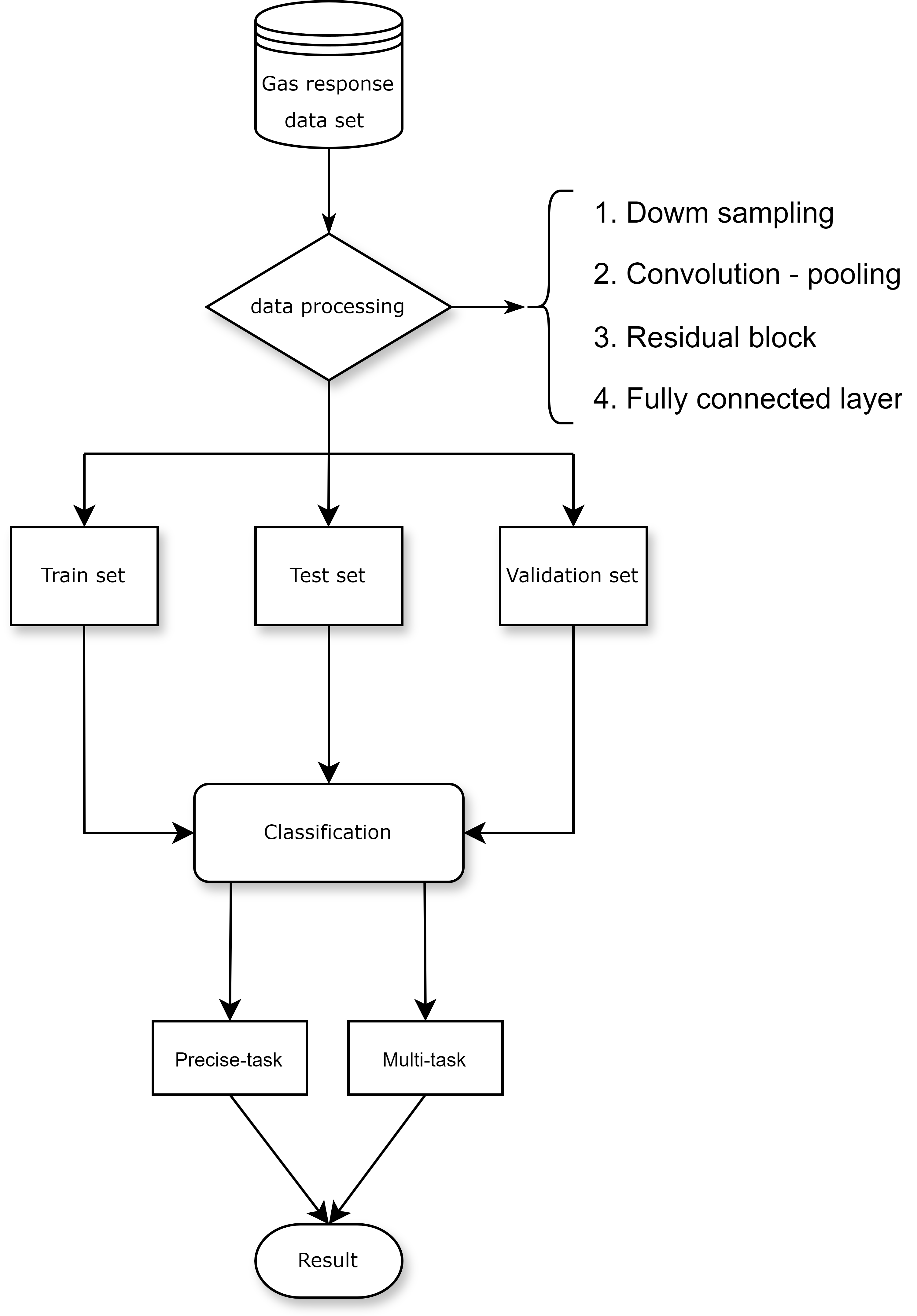
Figure S9.** Gas data features for 1D-ResNet deep learning process framework structure diagram.


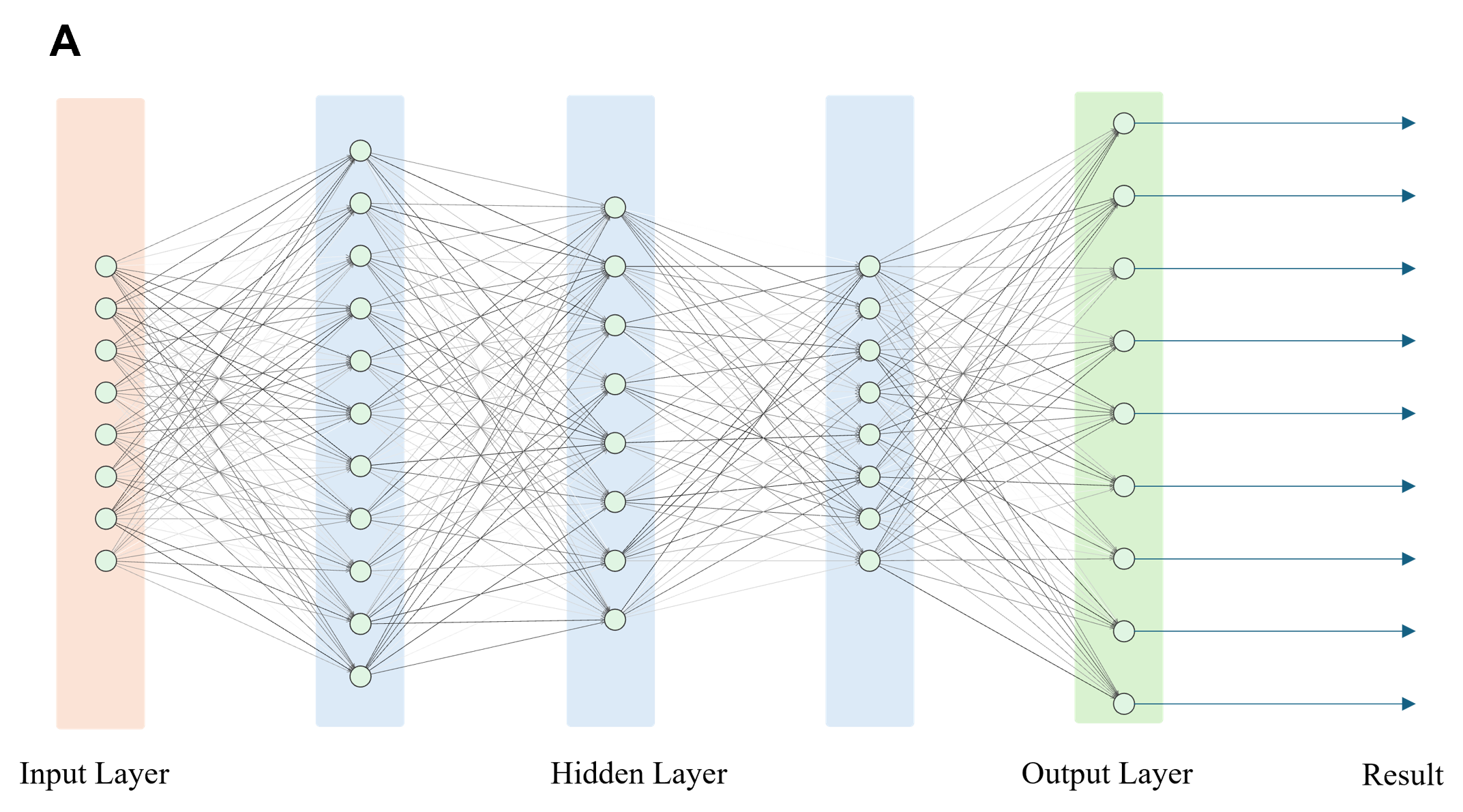


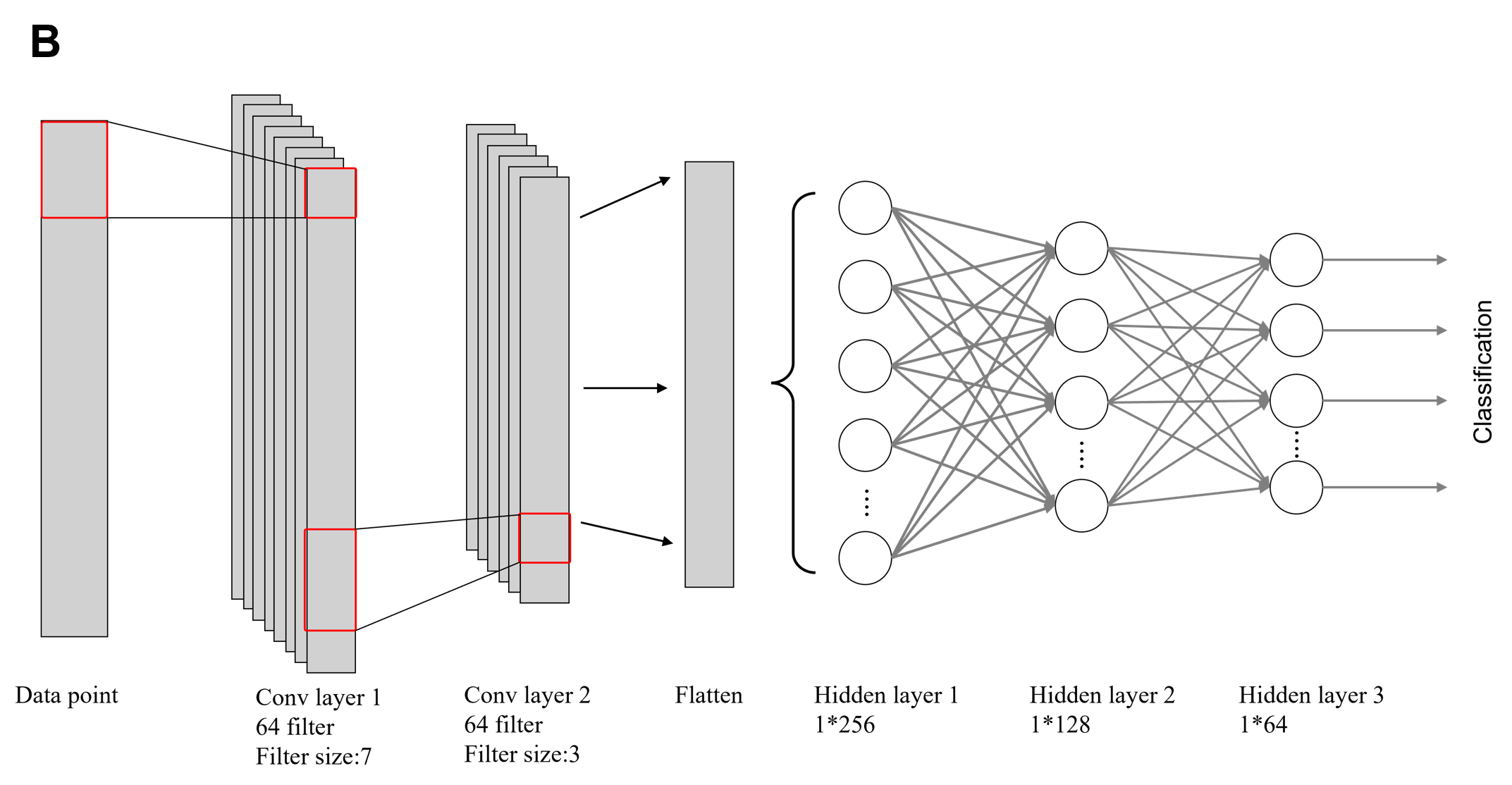


**Figure S10.** Classic deep learning model framework: (A) DNN and (B) 1D-CNN.


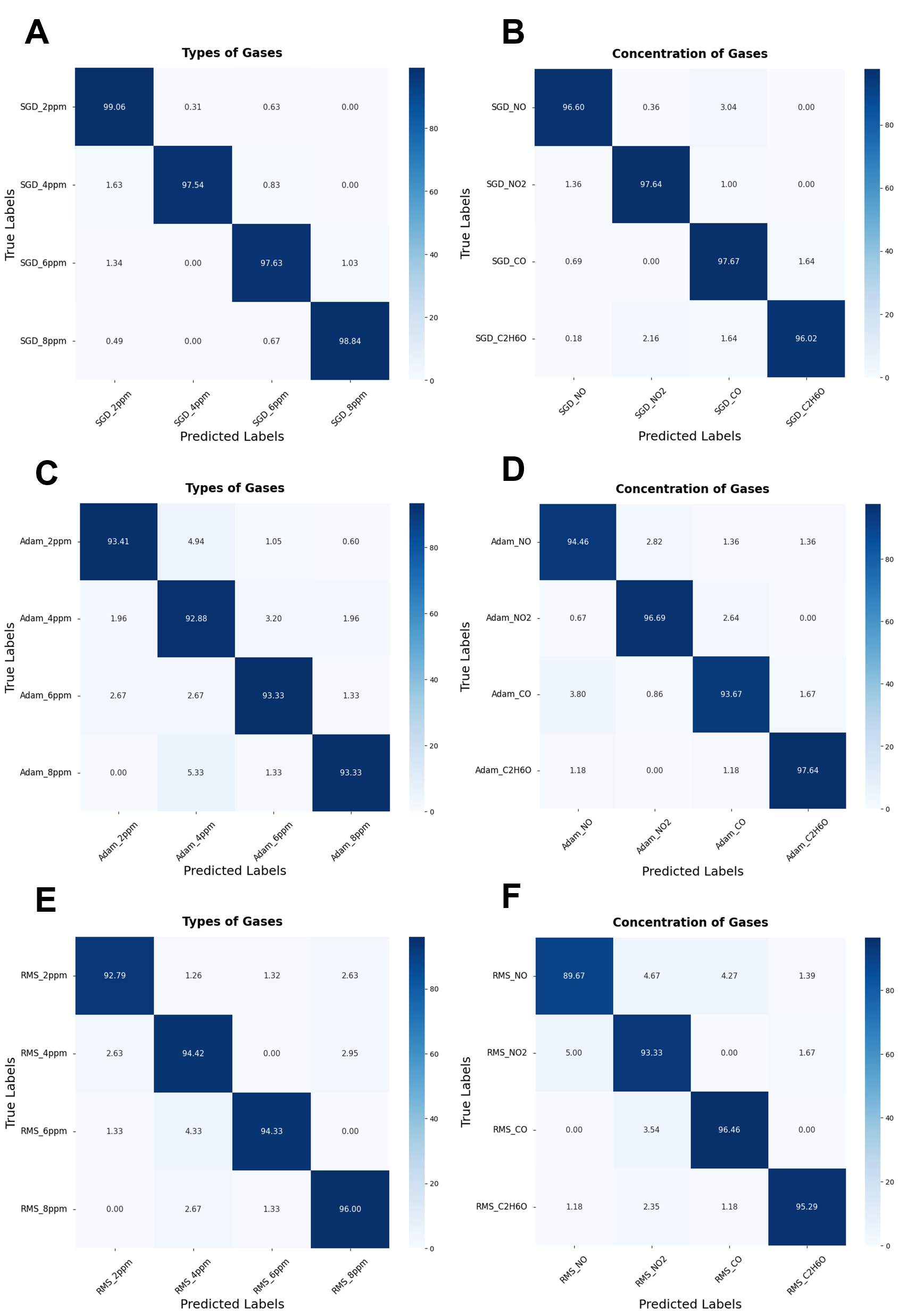


**Figure S11.** Confusion matrix results of multi-task classification (left: gas type, right: gas concentration) under three optimizers: (A, B) SGD; (C, D) Adam; (E, F) RMS-prop.


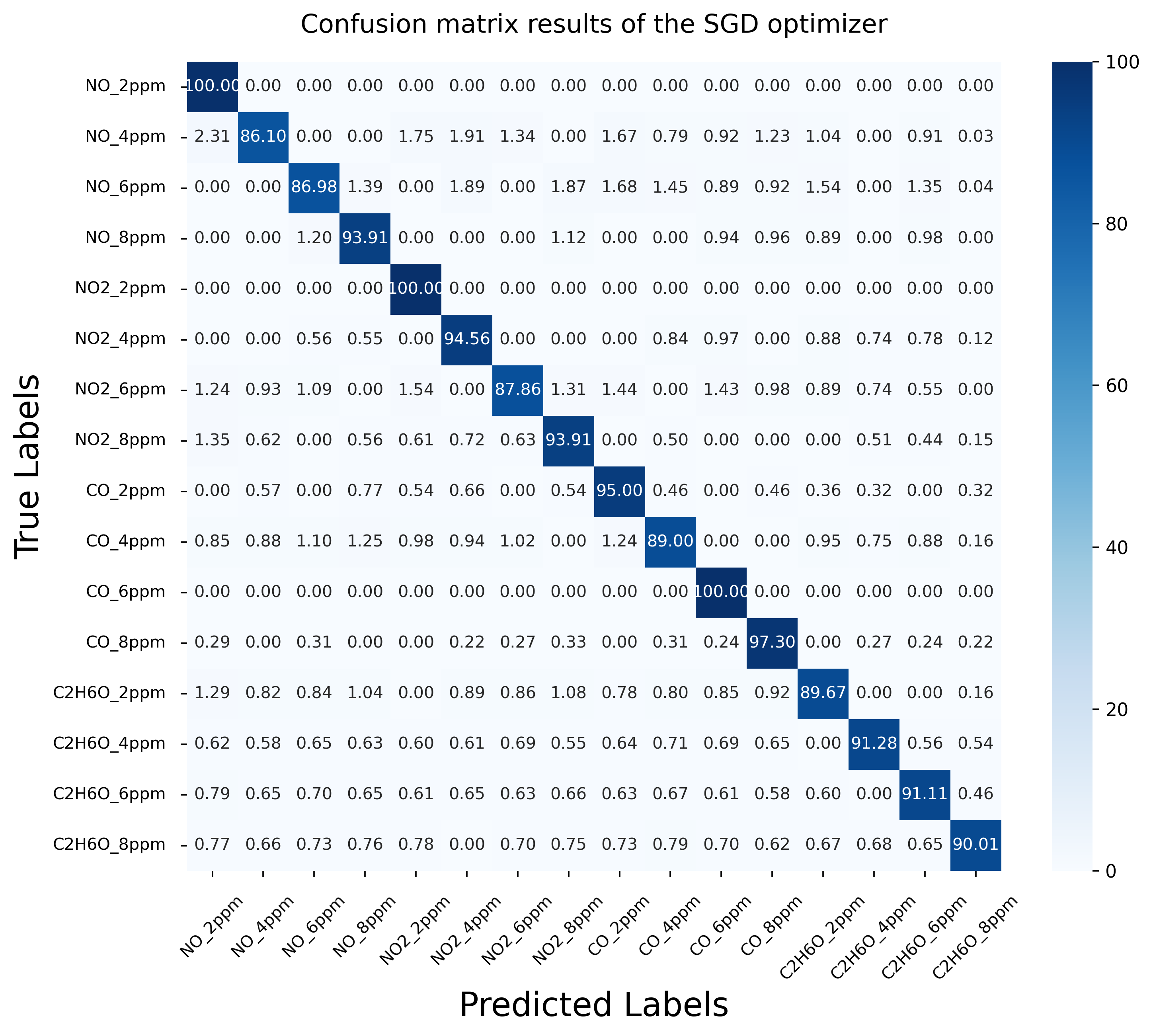


**Figure S12.** Confusion matrix results of the SGD optimizer for the precise classification task (92.92%).


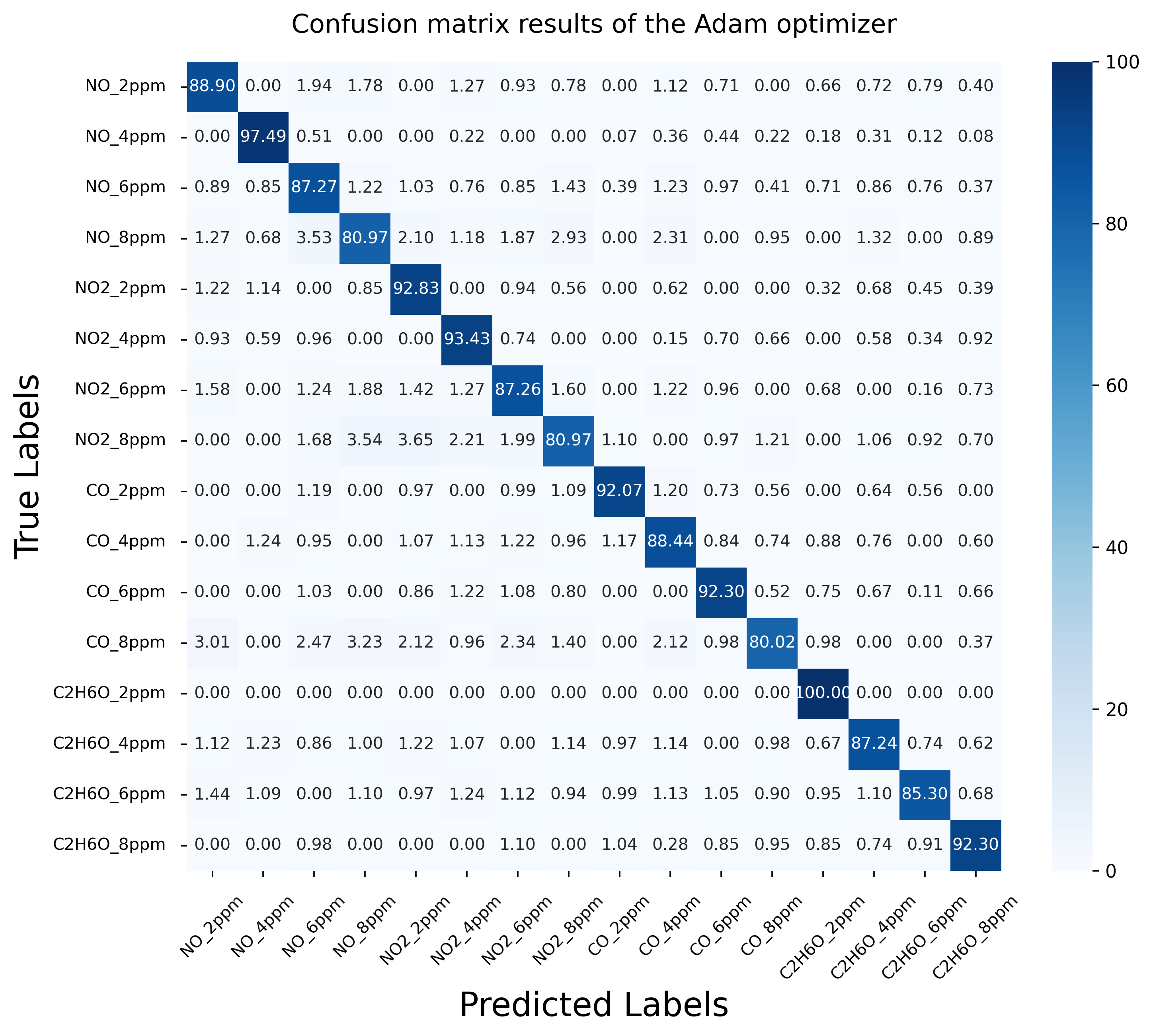


**Figure S13.** Confusion matrix results of an Adam optimizer for the precise classification task (89.17%).


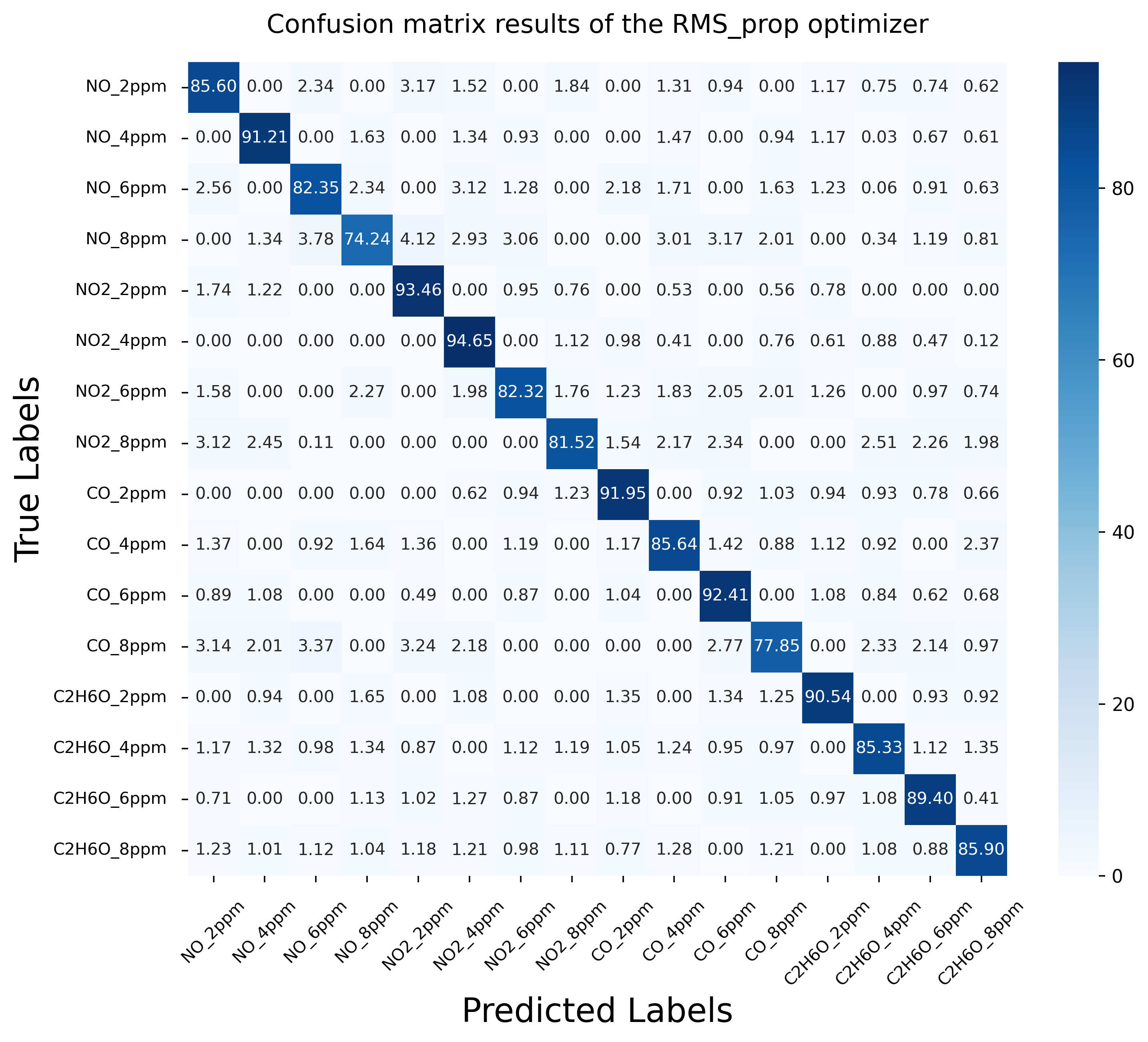


**Figure S14.** Confusion matrix results of the RMS-prop optimizer for the precise classification task (86.52%).


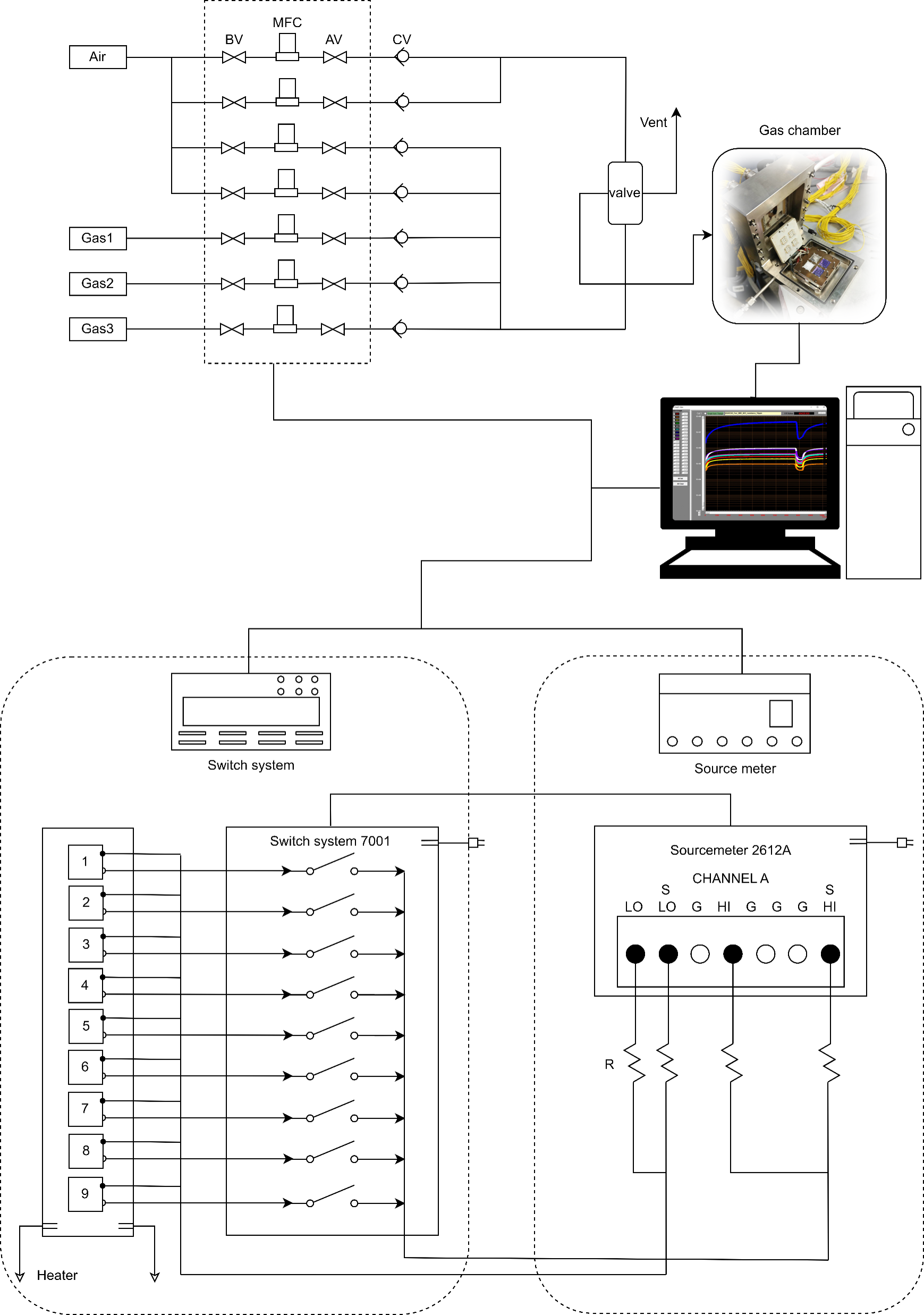


**Figure S15.** Gas measurement and monitoring setup. The gas supply module features one air flow path and three separate target gas flow paths, enabling target concentration adjustments by mixing air with the target gases. The measurement and monitoring setup supports up to four sensor array chips, enabling the simultaneous observation of gas responses across 36 channels.


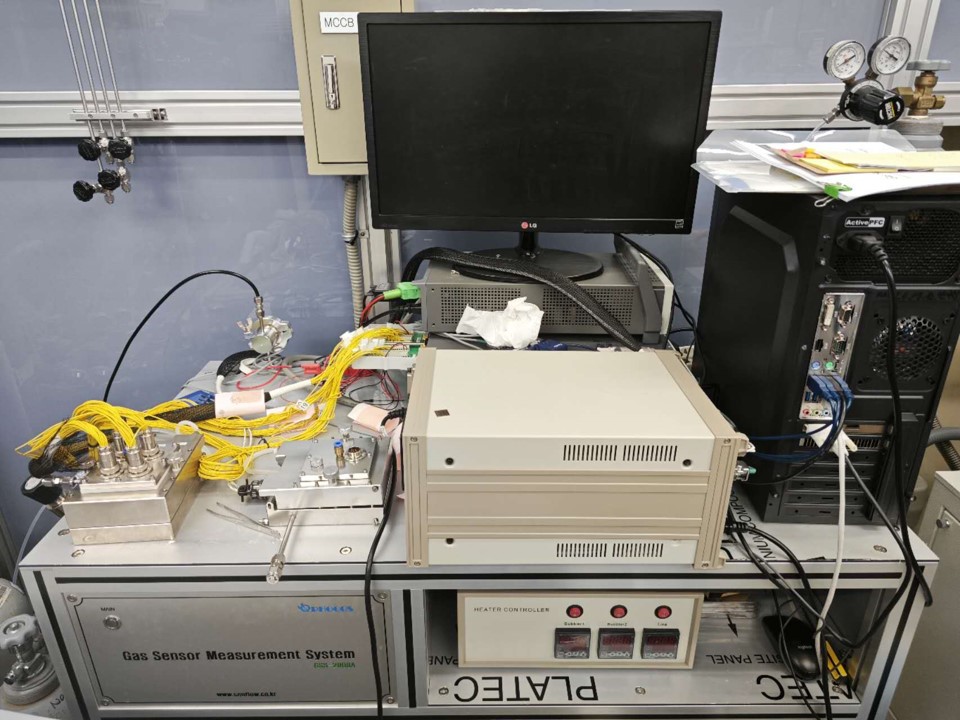

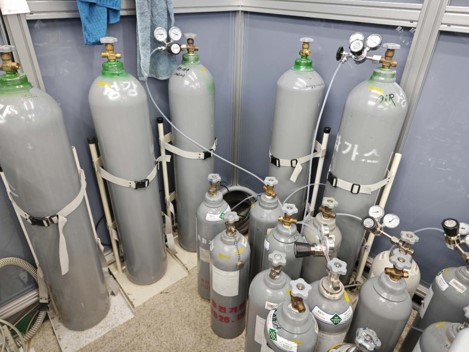


**Figure S16.** Schematic diagram of the actual experimental device.


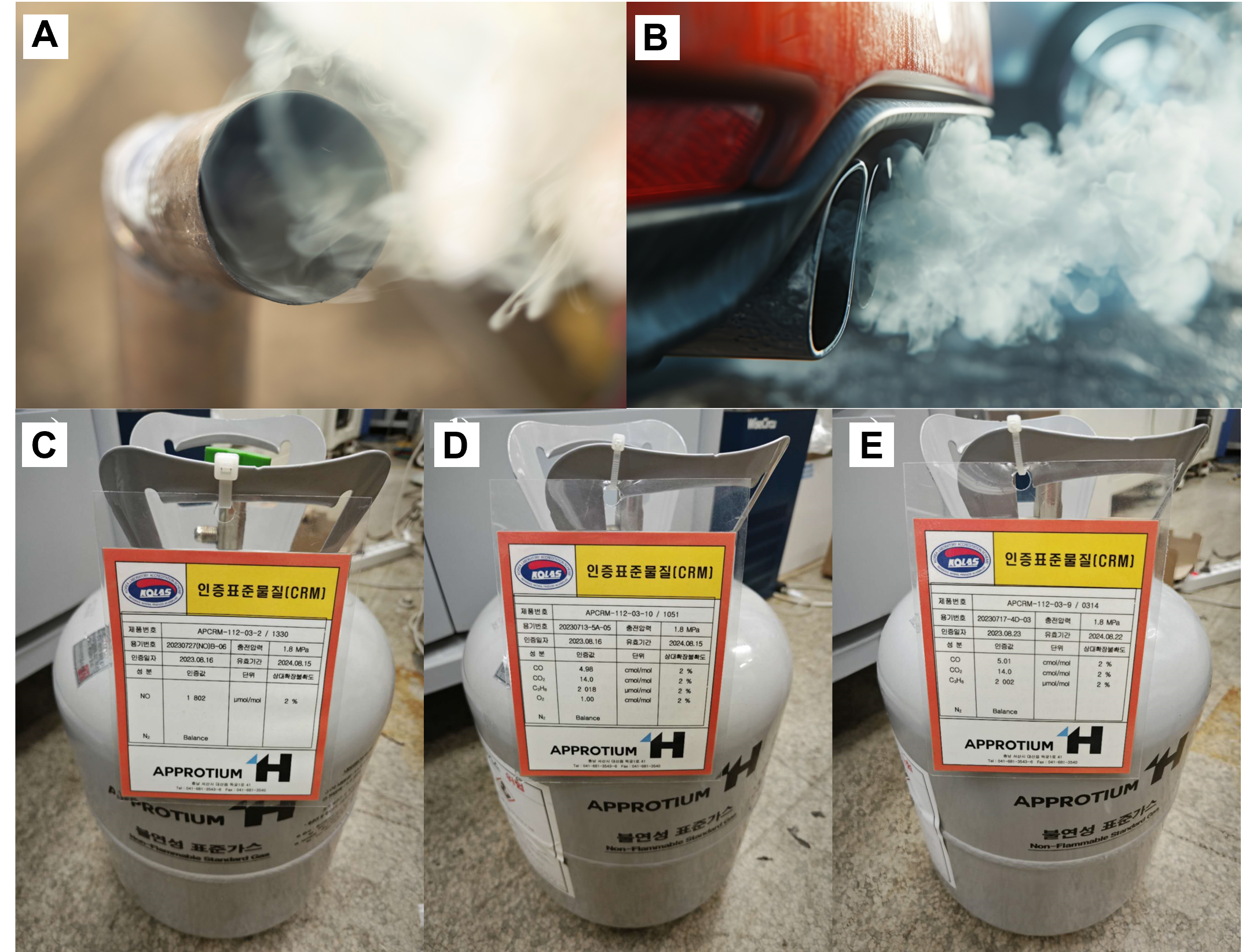


**Figure S17.** Vehicle exhaust emissions and certified reference materials for automobile exhaust gases, produced and validated through 195 rigorous and metrologically sound procedures by the Korean Laboratory Accreditation Scheme. (A) exhaust gases from a diesel-engine light-duty truck, (B) exhaust gases from a gasoline-engine car. (C, D) Standard exhaust gases for regulatory emissions testing of diesel engines, comprising NO (0.18%), CO (4.98%), CO_2_ (14.0%), C_3_H₈ (0.20%), and O_2_ (1%); (E) Standard exhaust gases for regulatory emissions testing of gasoline engines, comprising CO (5.01%), CO_2_ (14.0%), and C_3_H₈ (0.20%).


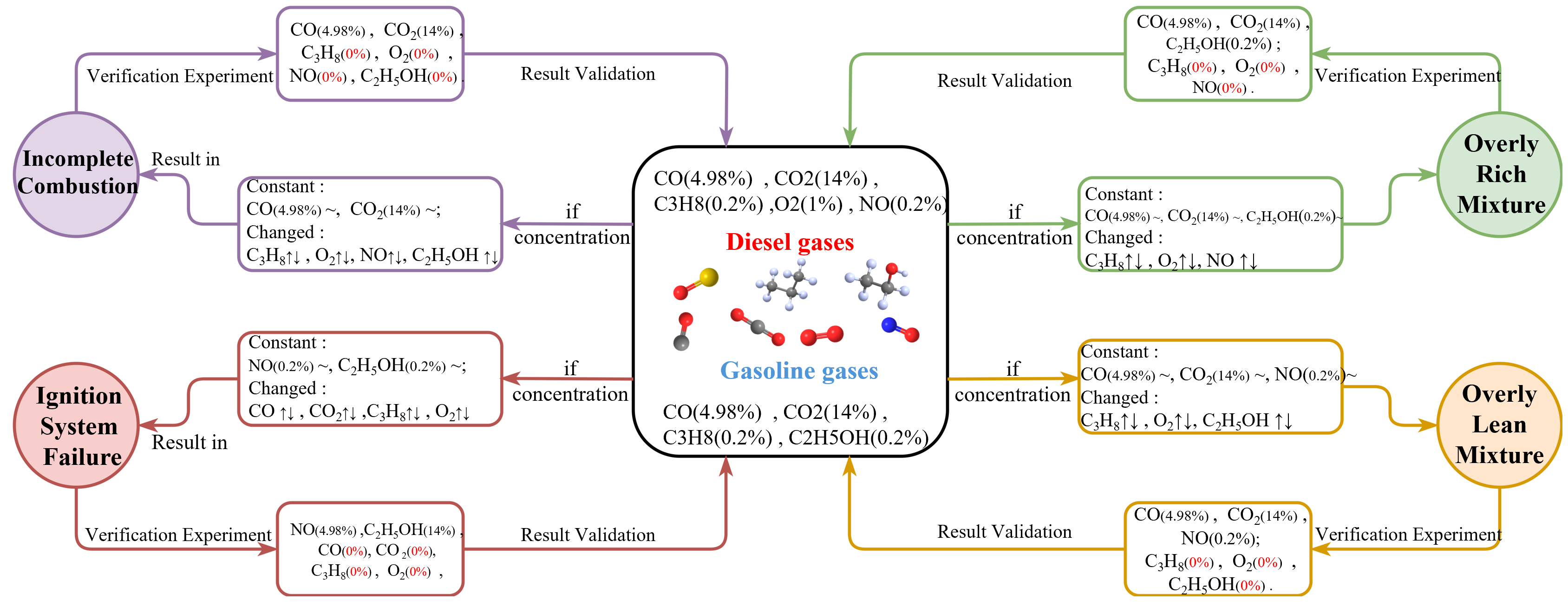


**Figure S18.** Schematic diagram of the gas composition changes under four typical engine faults.


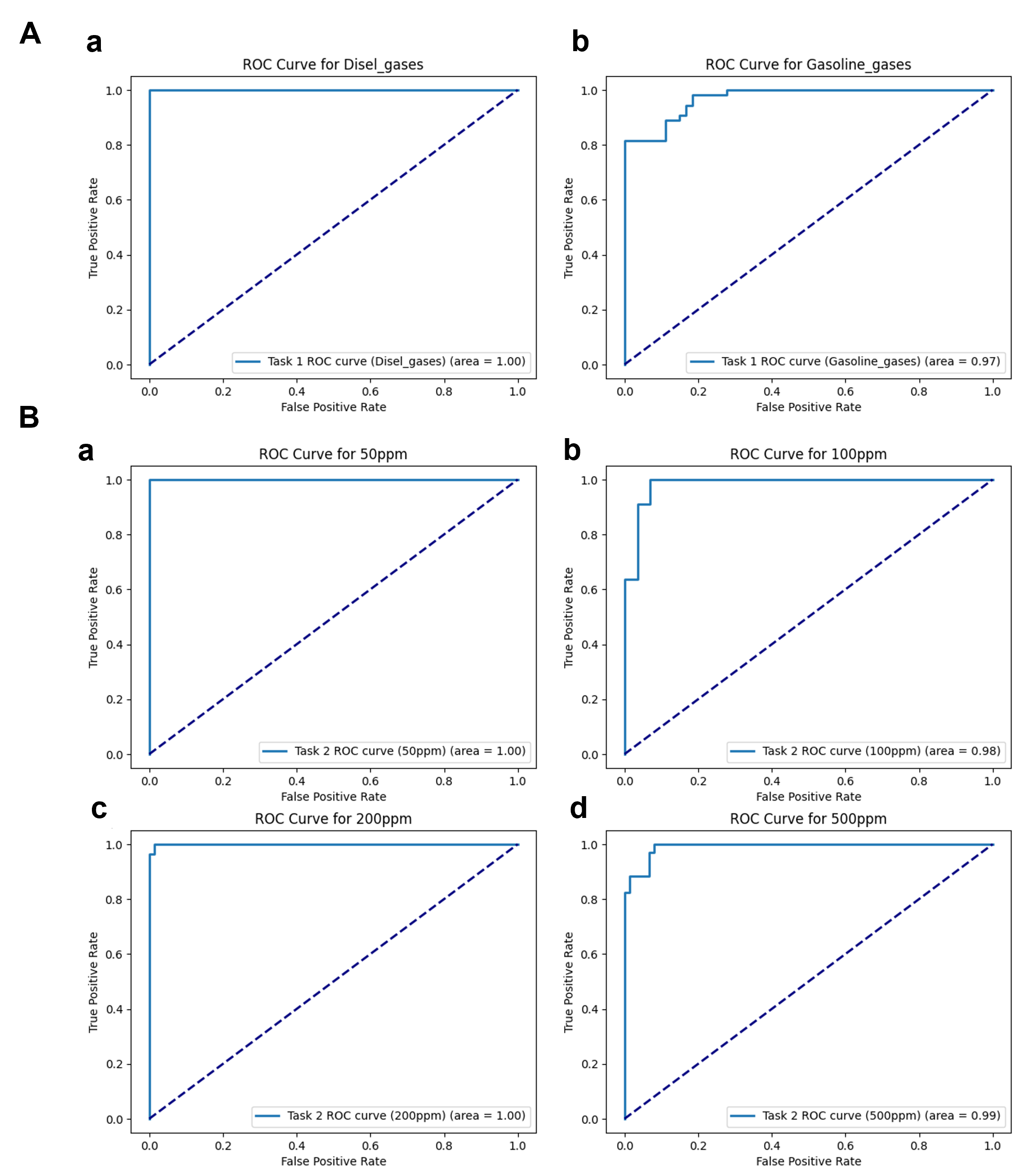


**Figure S19.** ROC/AUC evaluation results for multi-task classification of vehicle exhaust gases experiments: (A) gas type classification and (B) gas concentration classification.


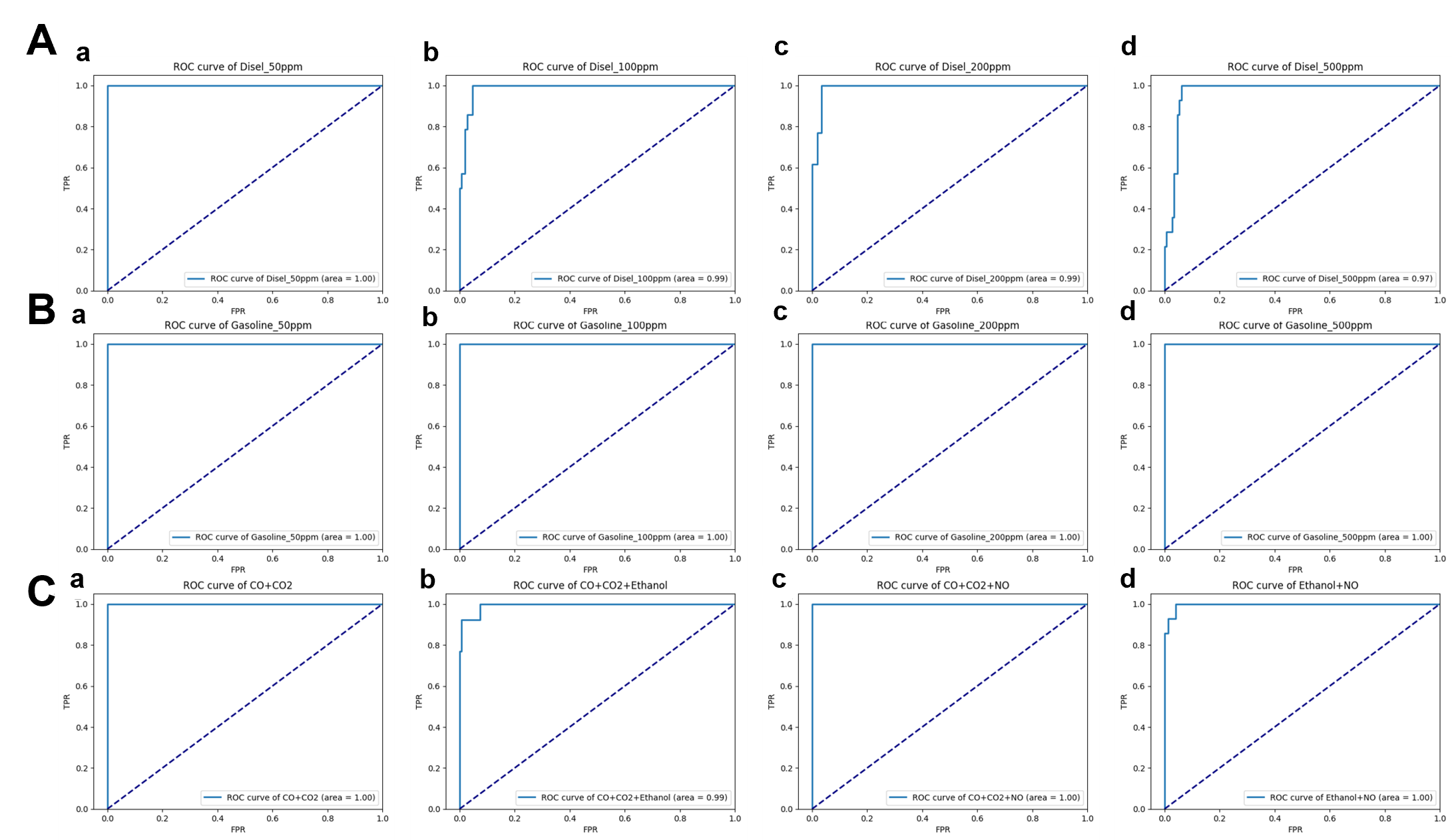


**Figure S20.** ROC/AUC evaluation results for precise-task classification of vehicle exhaust gases experiments: (A) diesel gas classification, (B) gasoline gas classification, and (C) Engine failure experiment classification.


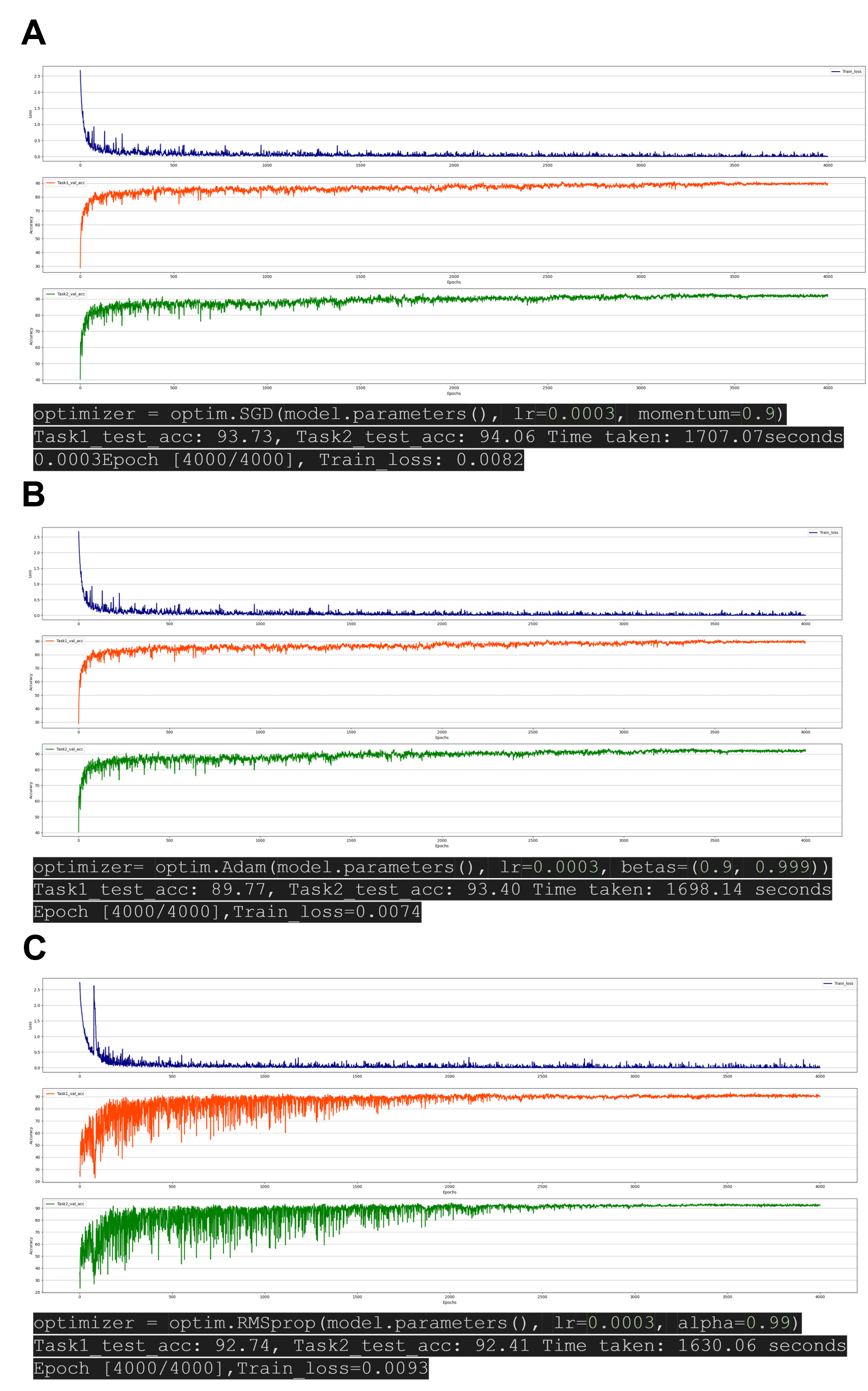


**Figure S21.** Loss and accuracy of the 1D-ResNet model for the multi-task classification with three different optimizers under four single gases (CO, NO_2_, and C_2_H_5_OH): (A) SGD, (B) Adam, and (C) RMS_prop.


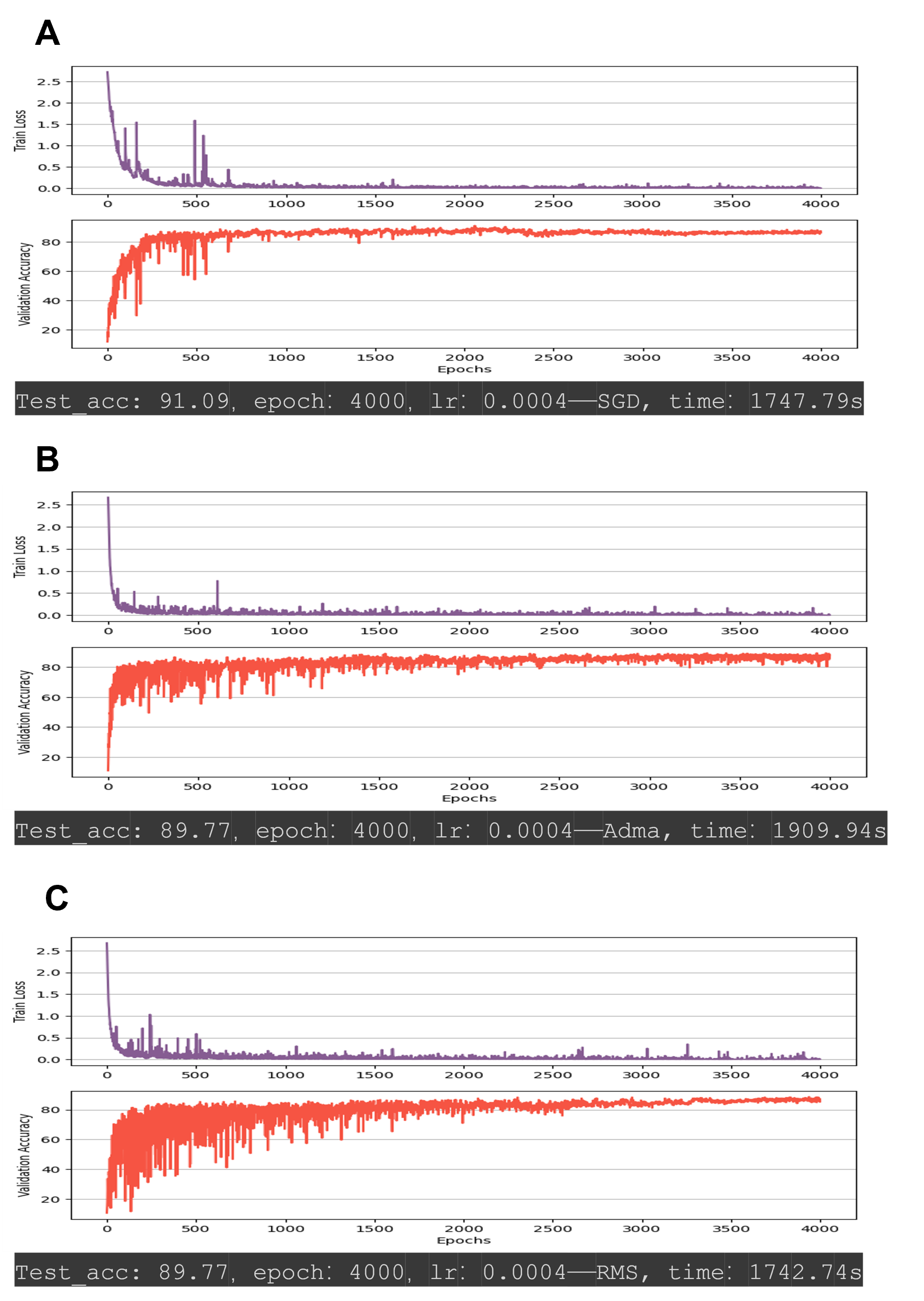


**Figure S22.** Loss and accuracy of the 1D-ResNet model for the precise-task classification with three different optimizers under four single gases (CO, NO, NO_2_, C_2_H_5_OH): (A) SGD, (B) Adam, and (C) RMS_prop.

**Table S1.** Comparison of the gas detection performance between previous studies using rGO or metal oxide materials and this study.

| Material | Maximum response | Temperature | Limit | Selectivity | Response time | Recovery time | Reference |
| --- | --- | --- | --- | --- | --- | --- | --- |
| rGO/tMO | 500% | 20°C | 2ppm | NO, NO_2_, CO, C_2_H_5_OH | 520s | 2000s | This work |
|  |  |  |  |  |  |  |  |
| rGO/MoS_2_ | 60% | 60°C | 2ppm | NO_2_ | 2000s | 2500s | ^[1]^ |
| PPy/Fe2O3 | 221% | 50°C | 10ppm | NO_2_ | 150s | 27s | ^[2]^ |
| rGO/SnO2 | 22% | 60°C | 1ppm | NO_2_ | 260 | 540 | ^[3]^ |
| 20ZNBV/rGO | 120% | 95°C | 1ppm | NO_2_ | 62.9 | 72.5 | ^[4]^ |
| CuO/ZnO/Al_2_O_3_ | 20% | 100°C | 0.25ppm | NO_2_ | 900s | 2000s | ^[5]^ |
| rGO | 10% | 100°C | 10ppm | NO, H_2_ | 600s | 300s | ^[6]^ |
| Sn-ZnO | 10% | 150°C | 1.5ppm | NO_2_ | 60s | 80s | ^[7]^ |
| rGO/NiO/ZnO | 80% | 140°C | 2ppm | NO_2_ | 39s | 16s | ^[8]^ |
| CuO/ZnO | 73% | 200°C | 100ppm | NO_2_ | 5s | 280s | ^[9]^ |
| SnO2 | 27% | 180°C | 100ppm | C_2_H_5_OH | 4s | 2s | ^[10]^ |
| ZnO/Ag | 85% | 225°C | 2000ppm | C_2_H_5_OH | 50s | 100s | ^[11]^ |
| Fe2O3/SnO2 | 50% | 650°C | 10ppm | NO_2_ | 25s | 80s | ^[12]^ |
| Co3O4 | 7% | 250°C | 50ppm | CO | 4s | 6s | ^[13]^ |
| ZnO | 16% | 300°C | 100ppm | CO | 25s | 35s | ^[14]^ |
| Au/ZnO | 40% | 350°C | 100ppm | CO | 400s | 500s | ^[15]^ |
| Ag/ZnO | 27% | 300°C | 50ppm | C_2_H_5_OH | 20s | 60s | ^[16]^ |

**Table S2.** Parameters and results of multi-task classification in vehicle exhaust simulation experiments of three deep learning models.

| Model | Optimizer | Accuracy (%) (task1) | Accuracy (%) (task2) | Training time(s) | Epoch | Loss |
| --- | --- | --- | --- | --- | --- | --- |
| DNN | SGD | 96.30 | 79.63 | 70.45 | 120 | 0.6884 |
|  | Adam | 97.22 | 92.01 | 73.42 | 150 | 0.5240 |
|  | Rms-prop | 94.44 | 87.04 | 68.58 | 150 | 0.4508 |
| 1D-CNN | SGD | 100.0 | 94.44 | 16.63 | 150 | 0.3371 |
|  | Adam | 100.0 | 95.37 | 16.26 | 150 | 0.3447 |
|  | Rms-prop | 100.0 | 94.15 | 16.38 | 150 | 0.0681 |
| 1D-ResNet | SGD | 100.0 | 99.07 | 22.51 | 120 | 0.1472 |
|  | Adam | 100.0 | 99.44 | 21.24 | 120 | 0.1483 |
|  | Rms-prop | 99.07 | 98.15 | 14.67 | 80 | 0.1085 |

**Table S3.** Parameters and results of the precise-task classification in vehicle exhaust simulation experiments of three deep learning models.

| Model | Optimizer | Accuracy (%) | Training time(s) | Epoch | Loss |
| --- | --- | --- | --- | --- | --- |
| DNN | SGD | 96.91 | 45.52 | 400 | 0.1053 |
|  | Adam | 97.53 | 37.39 | 400 | 0.1053 |
|  | Rms-prop | 96.91 | 47.58 | 400 | 0.0167 |
| 1D-CNN | SGD | 95.68 | 101.60 | 800 | 0.0994 |
|  | Adam | 98.08 | 89.14 | 800 | 0.1061 |
|  | Rms-prop | 96.91 | 102.68 | 800 | 0.0014 |
| 1D-ResNet | SGD | 98.77 | 39.39 | 150 | 0.1109 |
|  | Adam | 98.77 | 41.17 | 150 | 0.0032 |
|  | Rms-prop | 98.77 | 39.33 | 150 | 0.0004 |

[1] Y. Zhou, G. Liu, X. Zhu, Y. Guo, *Sensors and Actuators B: Chemical* **2017**, 251, 280.

[2] C. Wang, M. Yang, L. Liu, Y. Xu, X. Zhang, X. Cheng, S. Gao, Y. Gao, L. Huo, *Journal of colloid and interface science* **2020**, 560, 312.

[3] X. Zhu, Y. Guo, H. Ren, C. Gao, Y. Zhou, *Sensors and Actuators B: Chemical* **2017**, 248, 560.

[4] S. Bai, K. Tian, N. Han, J. Guo, R. Luo, D. Li, A. Chen, *Inorganic Chemistry Frontiers* **2020**, 7, 1026.

[5] E. Di Francia, H. Guzmán, D. Pugliese, S. Hernández, J.-M. Tulliani, *Sensors and Actuators B: Chemical* **2024**, 420, 136456.

[6] N. Sharma, V. Sharma, S. Sharma, K. Sachdev, *Materials Letters* **2019**, 236, 444.

[7] S. T. Shishiyanu, T. S. Shishiyanu, O. I. Lupan, *Sensors and Actuators B: Chemical* **2005**, 107, 379.

[8] S. Bai, J. Han, J. C. Meng, L. Sun, J. Sun, Y. Zhao, P. Tang, R. Luo, D. Li, A. Chen, *Sensors and Actuators B: Chemical* **2021**, 339, 129720.

[9] S. M. Mali, S. S. Narwade, Y. H. Navale, S. B. Tayade, R. V. Digraskar, V. B. Patil, A. S. Kumbhar, B. R. Sathe, *ACS omega* **2019**, 4, 20129.

[10] A. Alhadi, S. Ma, T. Yang, S. Pei, P. Yun, K. A. Abbakar, Q. Zhang, N. Ma, M. H. Balal, H. A. Hamouda, *Advances in Nanoparticles* **2021**, 10, 66.

[11] N. Tarwal, A. Rajgure, J. Patil, M. Khandekar, S. Suryavanshi, P. Patil, M. Gang, J. Kim, J. Jang, *Journal of materials science* **2013**, 48, 7274.

[12] A. Bhardwaj, I.-h. Kim, J.-w. Hong, A. Kumar, S.-J. Song, *Sensors and Actuators B: Chemical* **2019**, 284, 534.

[13] D. Patil, P. Patil, V. Subramanian, P. A. Joy, H. S. Potdar, *Talanta* **2010**, 81, 37.

[14] Y. Zeng, L. Qiao, Y. Bing, M. Wen, B. Zou, W. Zheng, T. Zhang, G. Zou, *Sensors and Actuators B: Chemical* **2012**, 173, 897.

[15] G. Neri, A. Bonavita, S. Galvagno, P. Siciliano, S. Capone, *Sensors and Actuators B: Chemical* **2002**, 82, 40.

[16] A. Hastir, N. Kohli, R. C. Singh, *Materials Today: Proceedings* **2017**, 4, 9476.
